# Asymmetric total synthesis strategies of halichlorine and pinnaic acid

**DOI:** 10.1039/d3ra06955a

**Published:** 2023-11-17

**Authors:** Lu Liu, Minghua Jiang, Qingkang Zhang, Hong Chen, Yifu Zhang, Jian Zhang

**Affiliations:** a School of Pharmacy, Gansu University of Chinese Medicine Lanzhou 730000 P. R. China zhangjian@gszy.edu.cn hanyuelongfei@qq.com; b Northwest Collaborative Innovation Center for Traditional Chinese Medicine Co-Constructed by Gansu Province & MOE of PRC Lanzhou 730000 P. R. China; c Key Laboratory of Chemistry and Quality for TCM of the College of Gansu Province Lanzhou 730000 P. R. China

## Abstract

Halichlorine and pinnaic acid are structurally related natural alkaloids isolated from different marine organisms. These two marine alkaloids bearing a 6-azaspiro[4.5]decane skeleton demonstrate a wide range of biological effects. It is this kind of unique structure and potentially valuable biological activity that have prompted strong synthetic interest, making it a research focus in recent years. Since the first total synthesis of halichlorine and pinnaic acid completed by Danishefsky's group, many groups have reported their outstanding synthesis methods especially the asymmetric synthesis strategies. This review summarizes the asymmetric synthesis strategies of halichlorine and pinnaic acid using a 6-azaspiro[4.5]decane skeleton as the key intermediate, which can provide some guidance for related work.

## Abstract 2

Halichlorine and pinnaic acids are structurally related to natural alkaloids isolated from different marine organisms. This review summarizes the asymmetric synthesis strategies of halichlorine and pinnaic acid using a 6-azaspiro[4.5]decane skeleton as the key intermediate.

## Graphical abstract

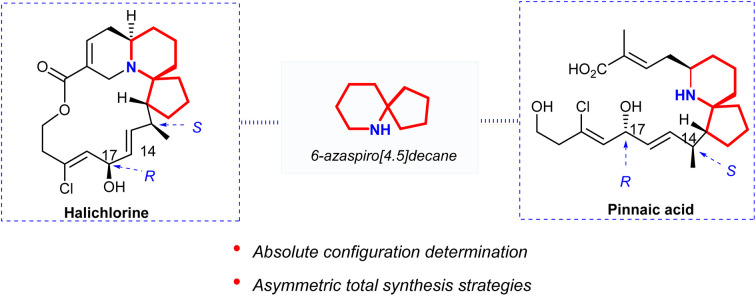

## 1 Introduction

**Fig. 1:**
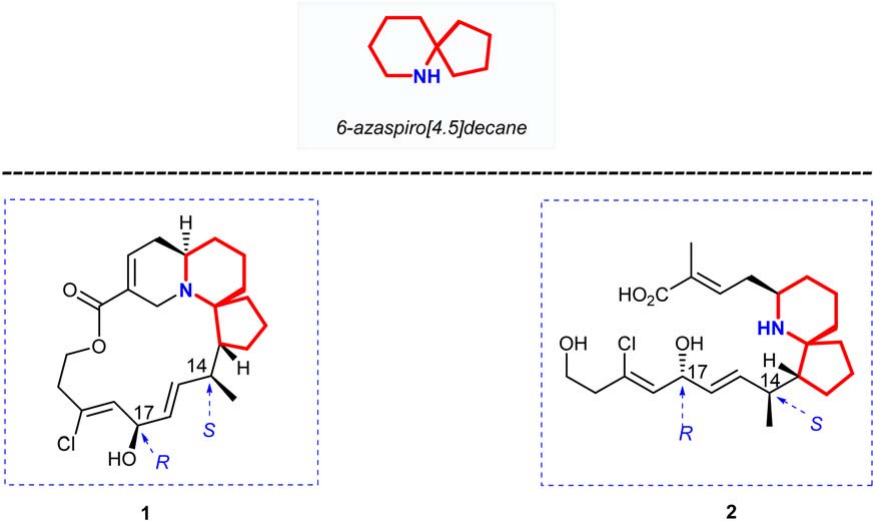
Structure of halichlorine (1) and pinnaic acid (2).

Halichlorine (1) and the pinnaic acid (2) are structurally related natural products isolated from different marine organisms (Fig. 1). The marine alkaloid halichlorine (1) was isolated from the black sponge *Halichondria okadai* Kadota by Uemure and co-workers in 1996.^1^ It selectively inhibits the expression of the inducible cell surface protein VCAM-1 (vascular cell adhesion molecule-1), and can be used to treat atherosclerosis, coronary artery disease, angina pectoris, and non-cardiovascular inflammatory diseases. Then Uemura's Laboratory isolated pinnaic acid (2) from the Okinawan bishell *Pinna muricata* in the same year, which was structurally closely related to halichlorine (1).^2^ Pinnaic acid (2) was found to inhibit cytoplasmic phospholipase A_2_(cPLA_2_) at a semi-inhibitory concentration of 0.2 mm *in vitro*. These two marine alkaloids possess significant biological properties and may have potential for use as biochemical tools or as leads for drug design.

The marine natural products halichlorine (1) and pinnaic acid (2) have attracted a lot of attention because of their good physiological activities and unique structures, and many research groups have carried out outstanding synthesis research on these two alkaloids. In 2005, Clive's group made a systematic and detailed summary on the synthesis of these two alkaloids.^3^ In recent years, lots of new asymmetric synthesis strategies of halichlorine (1) and pinnaic acid (2) have been reported. This review first summarizes the asymmetric synthesis strategies of halichlorine and pinnaic acid using 6-azaspiro[4.5]decane skeleton as the key intermediate over a span of approximately 20 years (1999–2022), which can provide some guidances for related workers. Furthermore, we also took into account the racemic synthesis work conducted between 2005 and 2022, which was not included in Clive's review. The constructions of 6-azaspiro[4.5]decane framework are the key steps of these asymmetric synthesis methods. In our efforts directed toward the synthesis of these two alkaloids base on the [2,3]-Stevens rearrangement, we have previously disclosed an efficient strategy for construction of the 6-azaspiro[4.5]decane skeleton.^4^

## 2 Absolute configuration determination and asymmetric synthesis of halichorine

### 2.1 Absolute configuration determination of halichorine

**Scheme 1:**
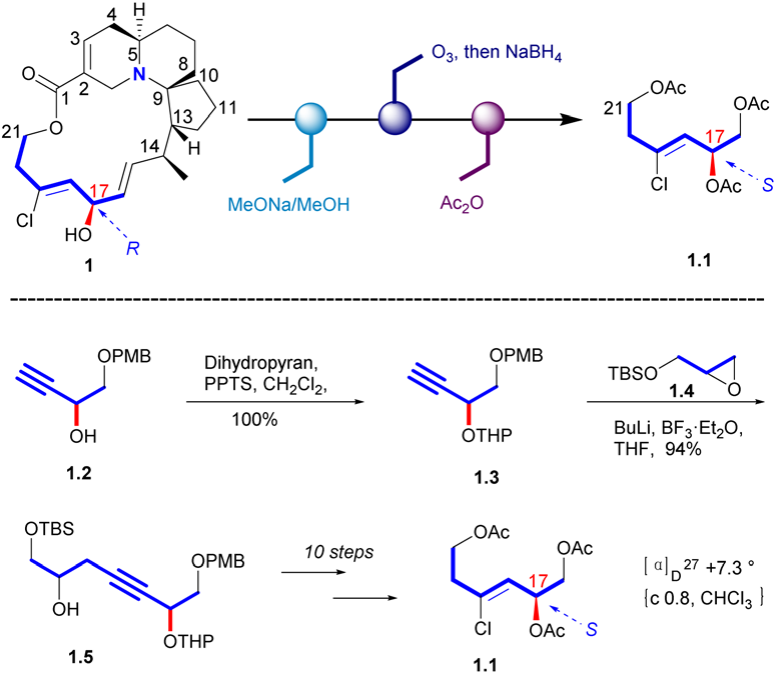
Confirmation of C17 stereochemistry of halichlorine.

In 1998, Uemura's Laboratory verified the absolute configuration of 1 through degradation studies (Scheme 1).^5^ Methanolysis of 1, followed by ozonolysis with reductive workup and global acetyl protection of the exposed alcohol functionalities yielded 1.1. The degradation product was compared with the sample prepared from a known alcohol 1.2, which it can be obtained from D-(+)-tartaric acid. The hydroxyl group of 1.2 was protected to a THP ether. Then the carbon chain extension product 1.5 was obtained in a high yield with the glycidyl ether 1.4. Then the final product (*S*)-triacetate 1.1 was obtained by the additional steps, whose NMR spectrum agreed with degradation product of natural halichlorine. It must be noted that the stereochemistry of C(17) is *S* in 1.1 but *R* in halichlorine, although there is no difference in the spatial arrangement of the key atoms. The enantiomer of 1.1 was made from l-tartaric acid, and HPLC analysis on a chiral column showed that 1.1 from d-tartaric acid corresponds to the degradation product from halichlorine, thereby establishing the absolute configuration of halichlorine, which was subsequently confirmed by the Danishefsky synthesis.^6^

### 2.2 Asymmetric synthesis of halichorine

**Scheme 2:**
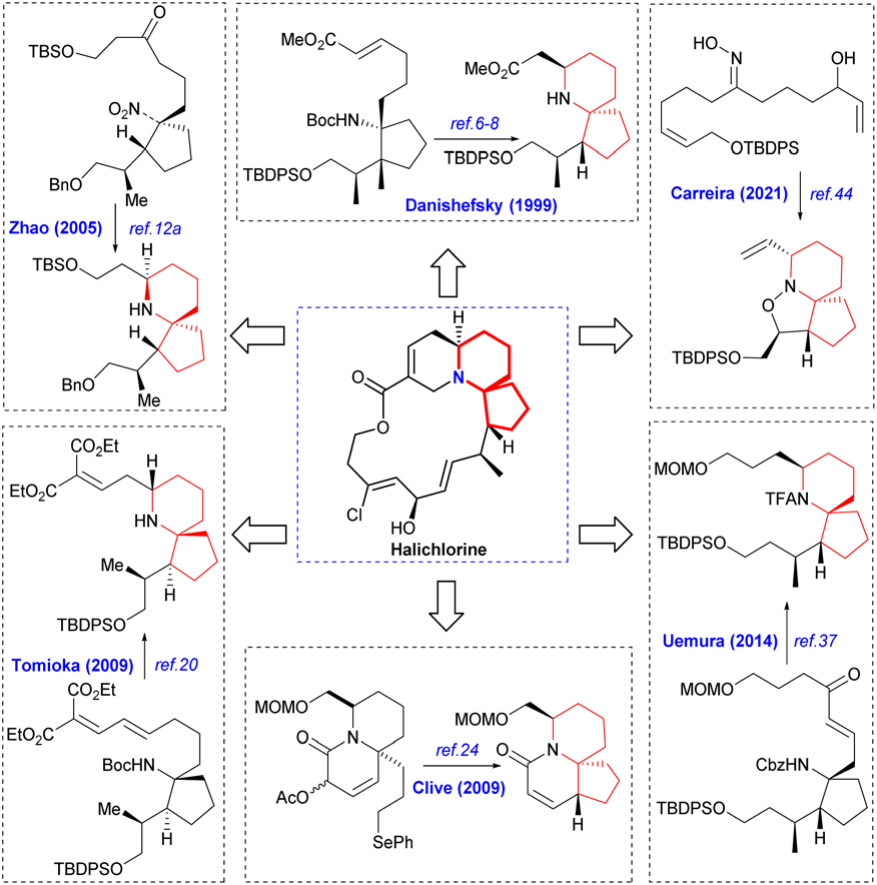
Asymmetric synthesis of halichorine *via* the 6-azaspiro[4.5]decane skeleton.

Since its initial proposal by Danishefsky in 1999, the synthesis strategy for halichlorine has attracted increasing attention from synthetic scholars. Over the years, numerous research groups have successfully completed the synthesis of halichlorine using various strategies. Each group's research work had their own characteristics in the synthesis strategies employed. We summarized the asymmetric synthesis strategies of halichlorine using 6-azaspiro[4.5]decane skeleton as the key intermediate, in which we can see the subtleties of the synthesis strategies of different research groups (Scheme 2).

#### 2.2.1 Danishefsky's first total synthesis of (+)-halichlorine

In 1999, Danishefsky's Laboratory completed the first asymmetric synthesis of halichlorine.^6–8^ The synthesis route started from the simple reaction of γ-keto acid 3.1 with D-(−)-phenylglycinol 3.2 to construct ‘Meyers lactam’ 3.3.^9^ Under the catalysis of Lewis acid, Meyers lactam reacted with allyl trimethylsilane to generate new lactam 3.4. Then, the nitrogen appendage was reduced and removed by the dissolving metal reduction, and the Boc group protection obtained compound 3.5. 3.6 was obtained by stereoselective methylation from the 3.5 convex structure by introducing C14 methyl.

**Scheme 3:**
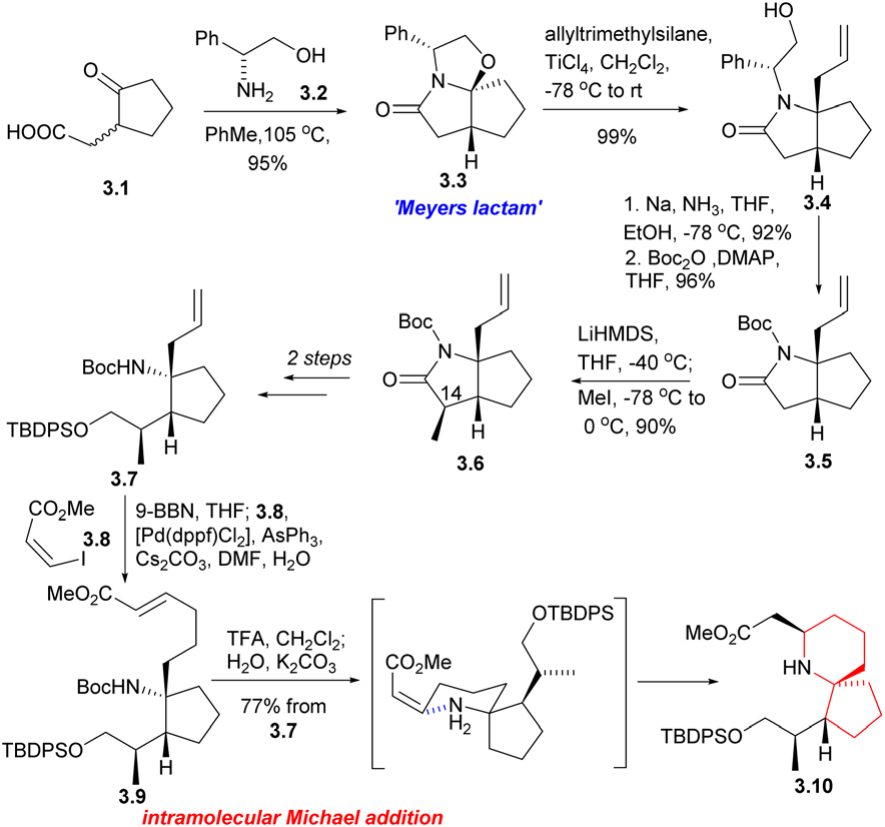
Danishefsky's synthesis of the 6-azaspiro[4.5]decane skeleton of halichlorine.

Then the compound 3.7 was obtained through multiple steps. Compound 3.7 was borohydrided with 9-BBN to obtain borane, and 3.8 was used as coupling agent to have Suzuki coupling reaction with it to extend the side chain (3.7 → 3.9). The Boc group was removed by TFA and then neutralized with K_2_CO_3_, resulting in spontaneous intramolecular Michael addition, providing the 6-azaspiro[4.5]decane skeleton with the desired configuration at C5 (3.9 → 3.10). This method is very effective to construct the 6-azaspiro[4.5]decane skeleton and the stereochemistry of the Michael addition presumably results from reaction *via* the conformation that with the larger substituent pseudoequatorial (Scheme 3).

**Scheme 4:**
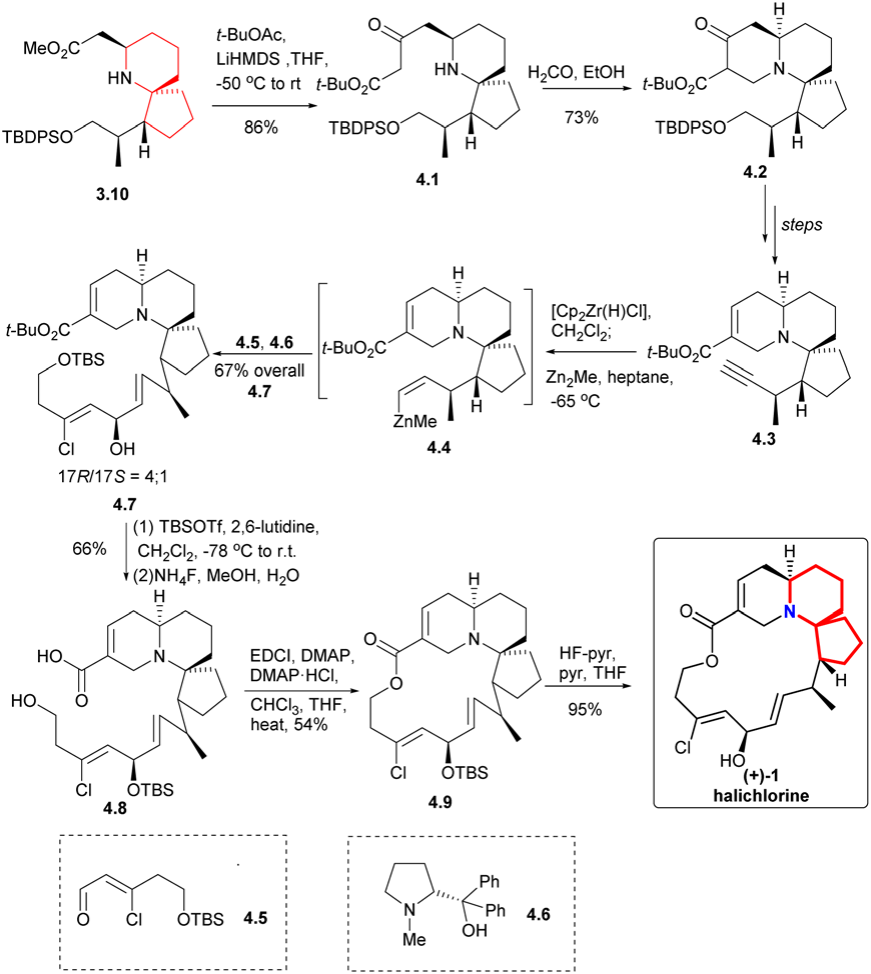
Danishefsky's synthesis of (+)-halichlorine.

Claisen condensation of 3.10 with *t*-BuOCOMe produced the β-keto ester 4.1 (Scheme 4), which was then subjected to Mannich reaction with formaldehyde to construct the A ring to obtain the compound 4.2. This process yielded the desired products as a mixture of diastereomer and tautomer. The compound 4.3 was obtained through multiple steps. The terminal alkyne was transformed by zirconation into organozirconium compounds, which were metallized with dimethylzinc to give compounds 4.4. The resulting zinc substance was successfully coupled with aldehyde 4.5, and the reaction was carried out in the presence of optical pure amino alcohol 4.6 (ref. 10) to obtain a 4 : 1 mixture of the required 17*R* epimer 4.7 and the corresponding 17*S* epimer (17*R*/17*S* = 4/1). Conversion of the *tert*-butyl ester was accomplished by the action of TBSOTf, which also protected the secondary alcohol.^11^ Then the TBS group was removed by NH_4_F in aqueous MeOH solution while leaving the protected secondary alcohol intact to obtained 4.8. At the same time, Keck macrocyclic esterification to form 17-OTBS-halichlorine (4.9), at staged the 17*R* and 17*S* series (8 : 1 mixture) which were separated, followed by desilylation to give (+)-halichlorine.

#### 2.2.2 Zhao and Ding's formal synthesis of halichlorine

**Scheme 5:**
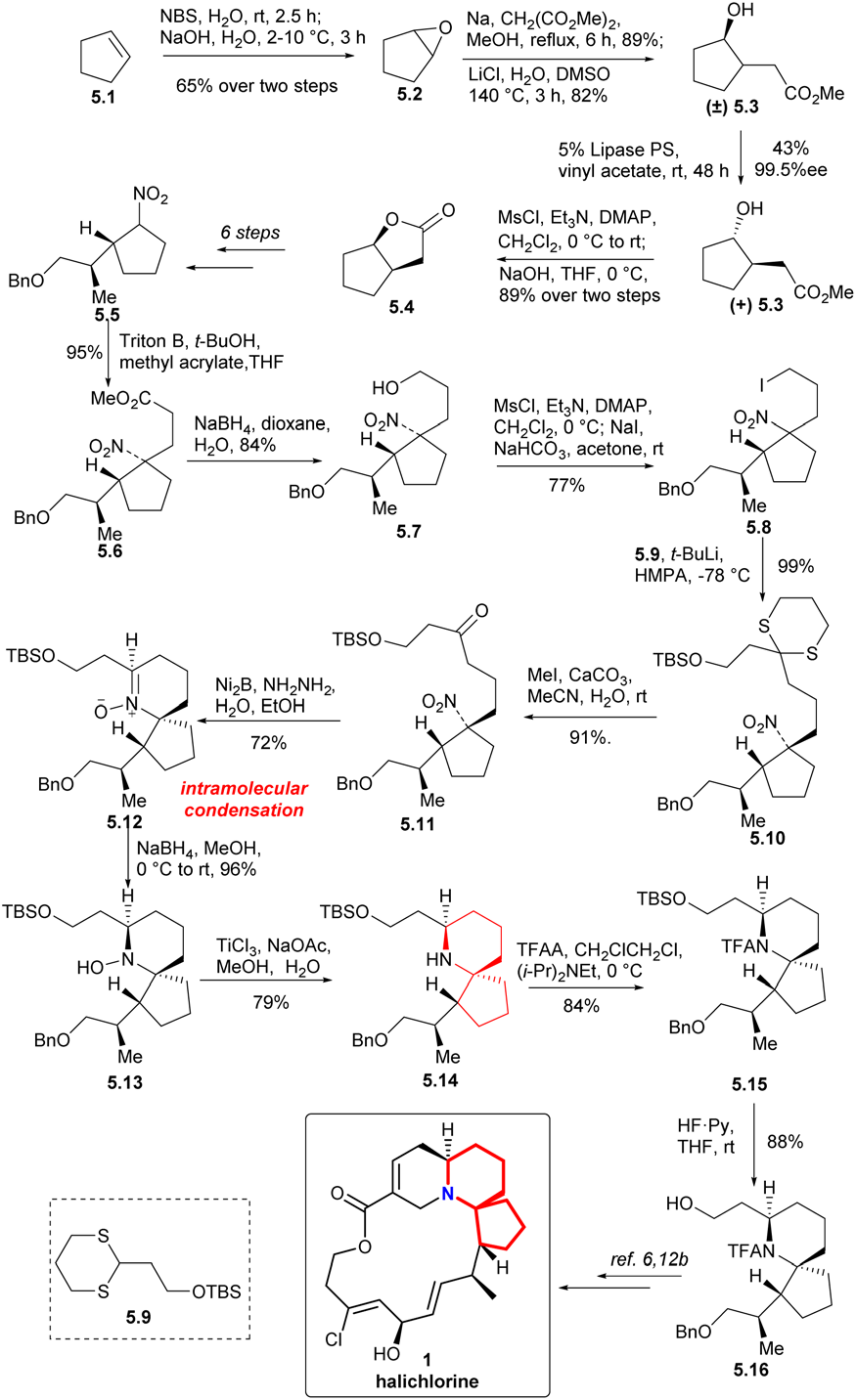
Zhao and Ding's formal synthesis of halichlorine.

In 2005, Zhao *et al.* constructed the 6-azaspiro[4.5]decane skeleton and achieved the formal synthesis of halichlorine (Scheme 5).^12*a*^ The cyclopentene 5.1 was treated with NBS in water and then with aqeous NaOH at 2–10 °C to afford the epoxide 5.2 in 65% yield. Epoxide was ring-opened with sodium malonic ester to obtain *trans*-diester and then decarboxlated to obtain the corresponding racemic acetates 5.3. Selective acylation of 5.3 under the control of Lipase PS in vinyl acetate produced the desired product (+) 5.3 (99.5% ee),^13^ which was converted to the bicyclolactone 5.4 through mesylation, hydrolysis, and cyclization. Nitro derivative 5.5 was obtained by multi-step transformations of 5.4. As a good donor, nitro derivative 5.5 underwent very efficient and stereoselective Micheal addition reaction with methyl acrylate. The addition product 5.6 was reduced with sodium borohydride to nitroalcohols 5.7, followed by iodization to form iodide 5.8. Dithione 5.9 was coupled with iodide 5.8 afforded 5.10,^14^ and then the thioketal hydrolysis to ketone obtained compound 5.11.

To form the 6-azaspiro[4.5]decane skeleton by the intramolecular condensation of δ-aminones, the *tert*-nitro in 5.11 must be reduced to an amino group. Ni_2_B-hydrazine combination was used to reduce the nitro group to obtain *N*-oxide 5.12.^15^ Reduction of the *N*-oxide 5.12 with NaBH_4_ (ref. 16 and 17*b*) followed by reduction of the hydroxyl group with TiCl_3_ (ref. 16*c* and 17) to obtain the 6-azaspiro[4.5]decane skeleton (5.12 → 5.13 → 5.14). Compound 5.16 was obtained by *N*-acylation with TFA and deprotection of TBS group (5.14 → 5.15 → 5.16). Since the intermediate 5.16 could be transformed to halichlorine,^6,12*b*^ so the formal synthesis of halichlorine was achieved.

#### 2.2.3 Tomioka's formal synthesis of (−)-halichlorine

In 2009, the Tomioka laboratory provided a method to efficiently construct the C9, C13, and C14 contiguous stereogenic centers of the key intermediates 6.8 through tandem conjugate addition–alkylation reaction.^18,19^ The tricyclic core structure was constructed successfully, and the formal synthesis of (−)-halichlorine was realized.^20^

**Scheme 6:**
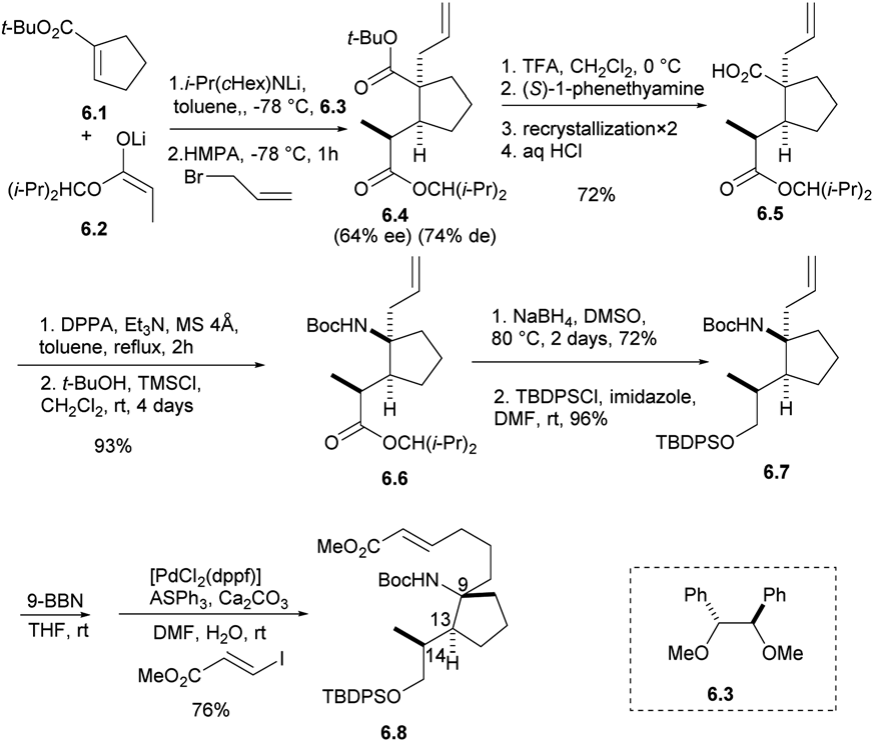
Tomioka's formal synthesis of (−)-halichlorine.

The synthetic strategy started with the addition of the lithium enolate 6.2 of propionate to 6.1 that would proceed by keeping the methyl group of 6.2 away from the cyclopentene moiety of 6.1 to give enolate intermediate, whose allylation was expected to proceed trans to the introduced propionate giving adduct 6.4.^21^ After the formation of carboxylic acid with TFA, carboxylic acid 6.5 was obtained by optical resolution of (*S*)-1-phenethylamine, and isocyanate was obtained by Curtius rearrangement with DPPA,^22^ which was inert to a nucleophilic addition of *t*-BuOH under refluxing conditions. The addition of TMSCl was effective to give Boc-amide 6.6 in 93% yield from 6.4 in two steps. The ester group was reduced by NaBH_4_ in DMSO and protected by TBDPSCl to obtain the intermediate 6.7.^7,8^ The stereochemistry of 6.7 was confirmed by spectroscopic data and the specific rotation of 6.8, which was prepared by hydroboration of 6.7 followed by the Suzuki coupling reaction, to be opposite that of natural halichlorin (Scheme 6).

**Scheme 7:**
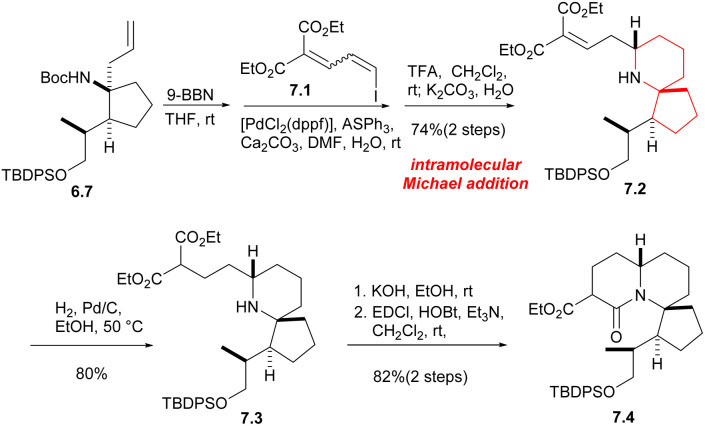
Construction of the tricyclic core of halichlorine by Tomioka.

Next, from the compound 6.7, the Tomioka's group synthesized the tricyclic core of halichlorine by a new strategy. Compound 6.7 hydroboride underwent a Suzuki coupling reaction with dienyl iodide 7.1, followed by the removal of the Boc group, gave the intramolecular Michael addition product 7.2 in 74% yield in two steps. The next stage was the formation of amides between the secondary amine and the ester moiety. After reduction of the double bond, the monosaponification product was obtained by KOH treatment and subsequent condensation of the amine with the carboxylic acid gave the lactam 7.4 with a tricyclic core of halichlorine (Scheme 7).

#### 2.2.4 Clive's formal synthesis of (+)-halichlorine

In 2009, Clive’ s group synthesized racemic halichlorine based on the method of completely substituted asymmetric center and developed a new route to optically pure piperidines. On the basis of this route, the formal synthesis of (+)-halichlorine was realized.^23,24^

**Scheme 8:**
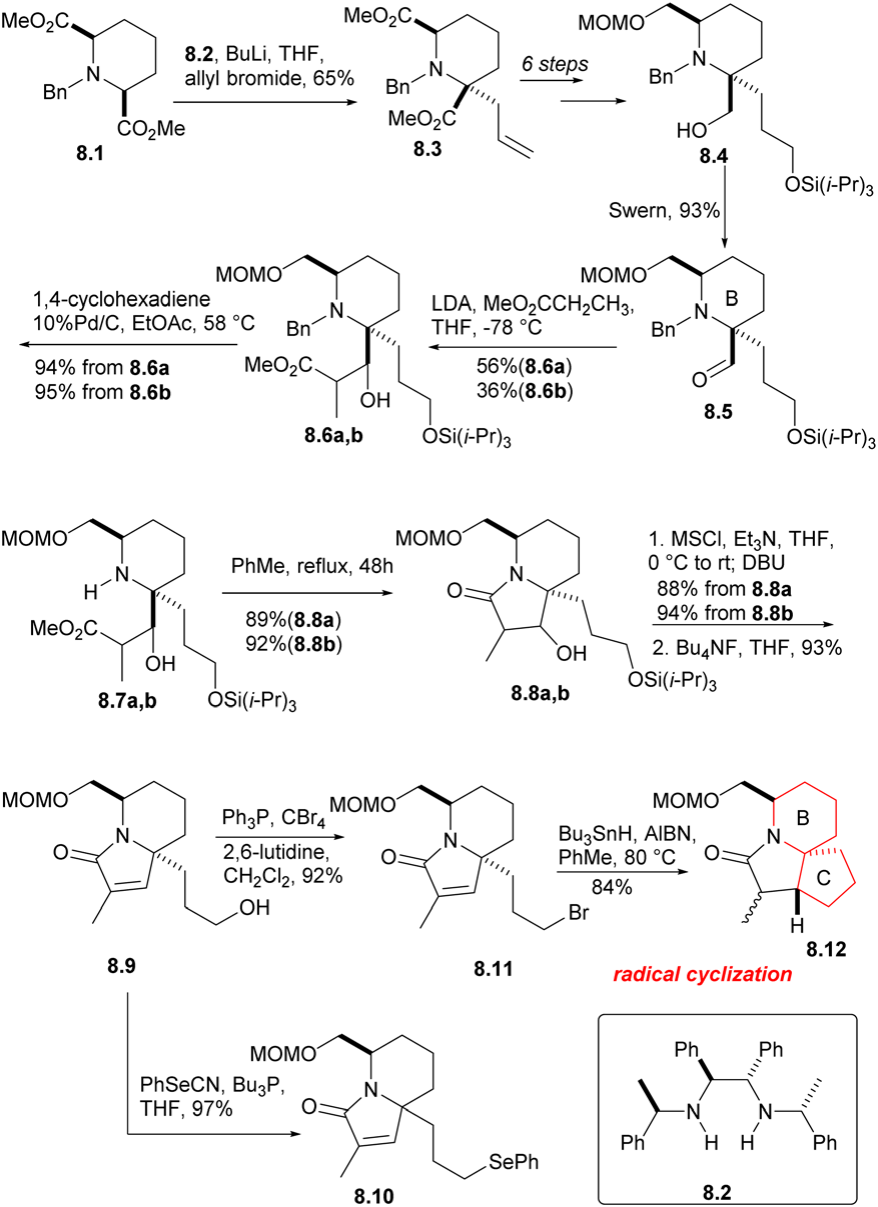
Synthesis of the 6-azaspiro[4.5]decane skeleton of halichlorine.

The *cis*-diester 8.1 was obtained from pyridine 2,6 dicarboxylic acid by esterification, hydrogenation and *N*-benzylation using the methods reported in the literature (Scheme 8). The symmetrical diester 8.1 was then subjected to asymmetric allylation (8.1 → 8.3), using the chiral base 8.2 (ref. 25 and 26) and allyl bromide. After a few simple steps, they got the compound 8.4, and Swern oxidation produced the expected aldehyde 8.5, which corresponds to the B ring of halichlorine and contains the required stereochemical and structural features. Aldol condensation of aldehyde 8.5 with methyl propionate yielded diastereomer alcohols of 8.6a and 8.6b, which were separable, but both can take subsequent reactions without further separation. The *N*-benzyl group was removed by heating with 10% Pd/C in the presence of 1,4-cyclohexadiene, and then the isomeric lactams were formed in the PhMe by heating the hydrogenolysis product (8.6a,b → 8.7a,b → 8.8a,b). The two lacamides 8.8a,b afforded the same unsaturated lactam 8.9*via* mesylation, prolonged heating with DBU in THF an desilication. The hydroxyl of 8.9 was replaced by bromine under the conditions of Ph_3_P/CBr_4_, then radical cyclization produced the desired the tricyclic lactam 8.12. However, the stereochemical result at C (17) was unfavorable, the β-isomer required for the main isomer (4 : 1) was the secondary component. Although this isomer ratio could be almost reversed by using the *t*-BuOK/*t*-BuOH equilibrium, the differential isomers were too difficult to separate. As a result, the team decided to utilize the phenylseleno group 8.10.

**Scheme 9:**
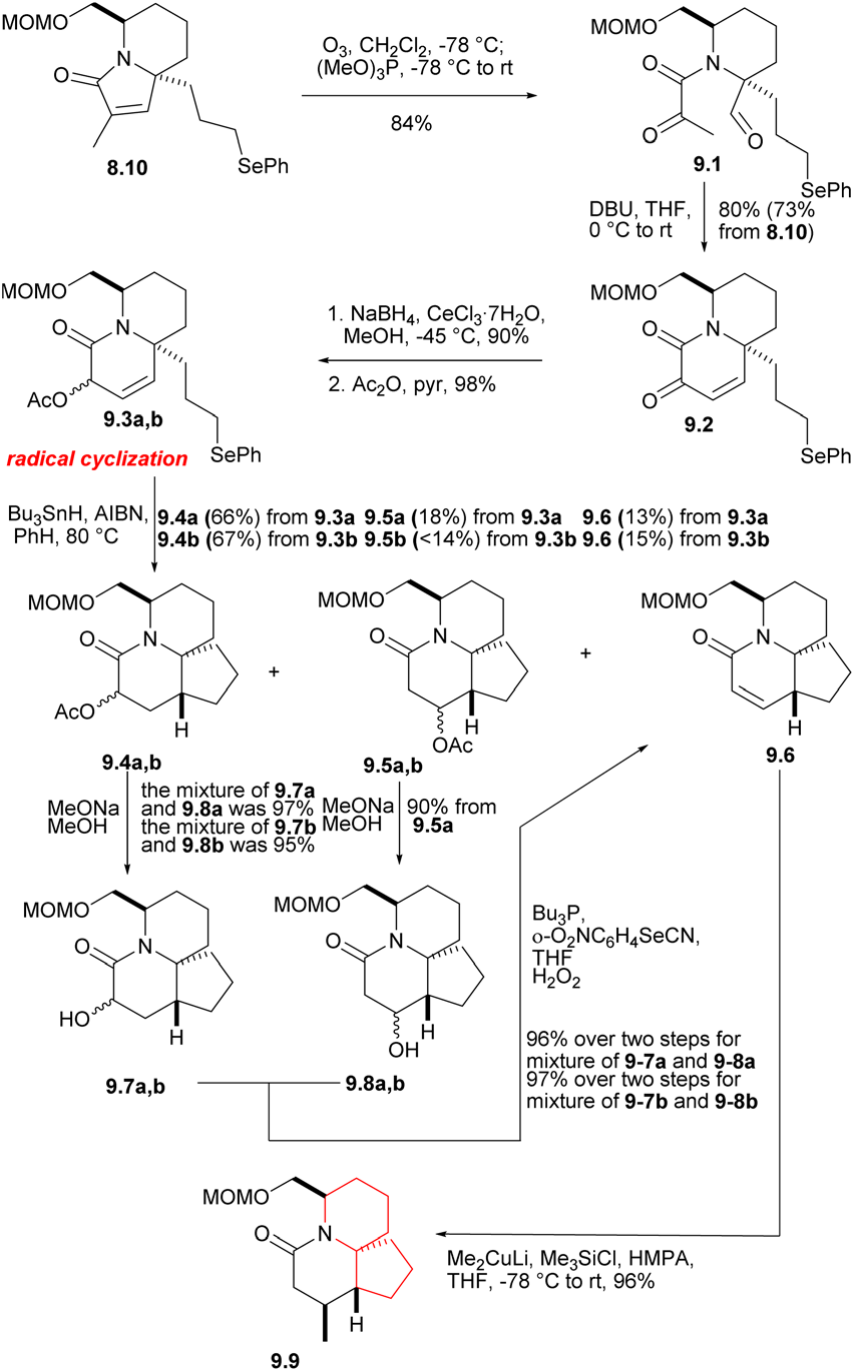
The exploration of ring C and introduction of the eventual C(17) methyl group.

The phenyl selenide 8.10 was subjected to ozonolysis and *in situ* reduction at a low temperature to give tricarbonyl selenide 9.1 (Scheme 9).^27^ After treatment with DBU, it underwent consecutive intramolecular aldol condensation and dehydration of hydroxylaldehydes (9.1 → 9.2), and was reduced to the corresponding α-hydroxy lactam under Luche condition,^28^ which reduced its reactivity. The hydroxyl group was then protected by acetylation to afford 9.3a,b. In the radical cyclization of 9.3a,b, the main products were the expected acetates 9.4a [AcO and C(17*a*)H *syn*] and 9.4b [AcO and C(17*a*)H *anti*], but the corresponding rearranged acetates^29^9.5a, 9.5b and the enone 9.6 were also isolated. 9.4 were hydrolyzed at a yield of at least 95% to obtain the corresponding alcohol (MeONa, MeOH), and these can be converted to 9.6, either by mesylation and base treatment, or treated with o-(O_2_N)C_6_H_4_SeCN and Bu_3_P,^30^ then oxidized with 30% H_2_O_2_. 9.5 were hydrolyzed and converted to 9.6, too. At this stage, they needed to introduce a methyl at the final C(17) position, and the reaction yield of conjugated addition^31^ of Me_2_CuLi and unsaturated lactam was very high, providing the product saturated lactam 9.9.

**Scheme 10:**
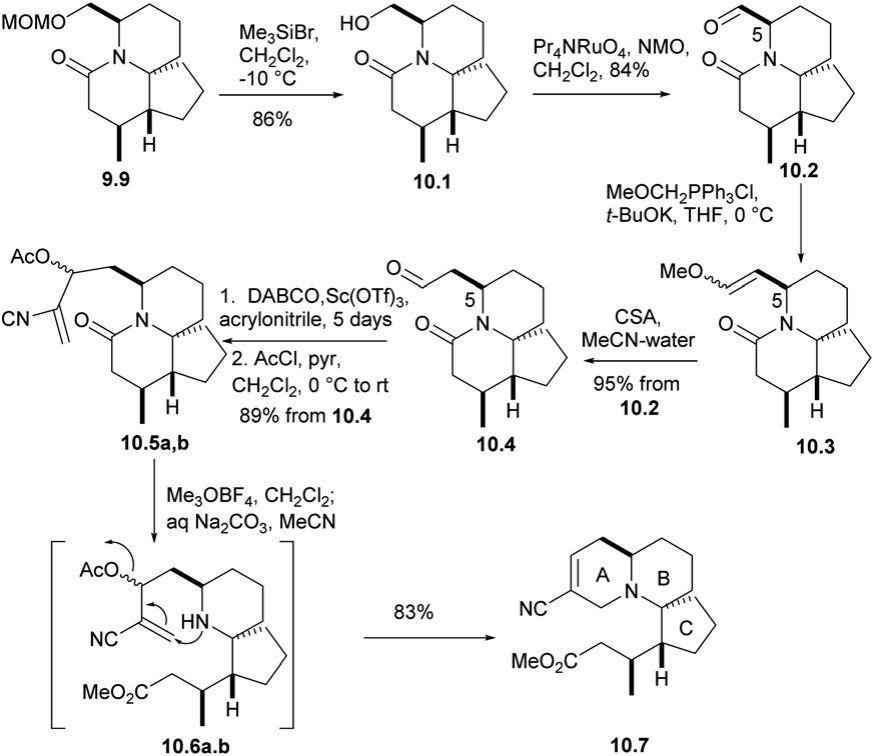
Construction of ring A.

Removing the MOM group of 9.9*via* Me_3_SiBr, *n*-PR_4_NRuO_4_, oxidizesing the primary alcohol to provide the corresponding aldehyde, Ph_3_P=CH(OMe) for Wittig olefination(9.9 → 10.1 → 10.2 → 10.3), and followed by acid hydrolysis afforded compound 10.4 (aldehyde 10.2 and the formation of enol ethers 10.3 did not cause any epimerization at C5). For the sake of constructing ring A, aldehyde 10.4 underwent a Baylis–Hillman reaction with acrylonitrile to provide the required alcohols at a high yield, and AcCl acetylation instantly converted these alcohols into the corresponding acetate 10.5a,b. With opening the lactam ring with Meerwein salt Me_3_O^+^BF_4_^−^, the resulting amines were subjected to spontaneous intramolecular conjugate displacement^32^ to unsaturated nitrile 10.7. At this point, the A, B, and C rings were constructed (Scheme 10).

**Scheme 11:**
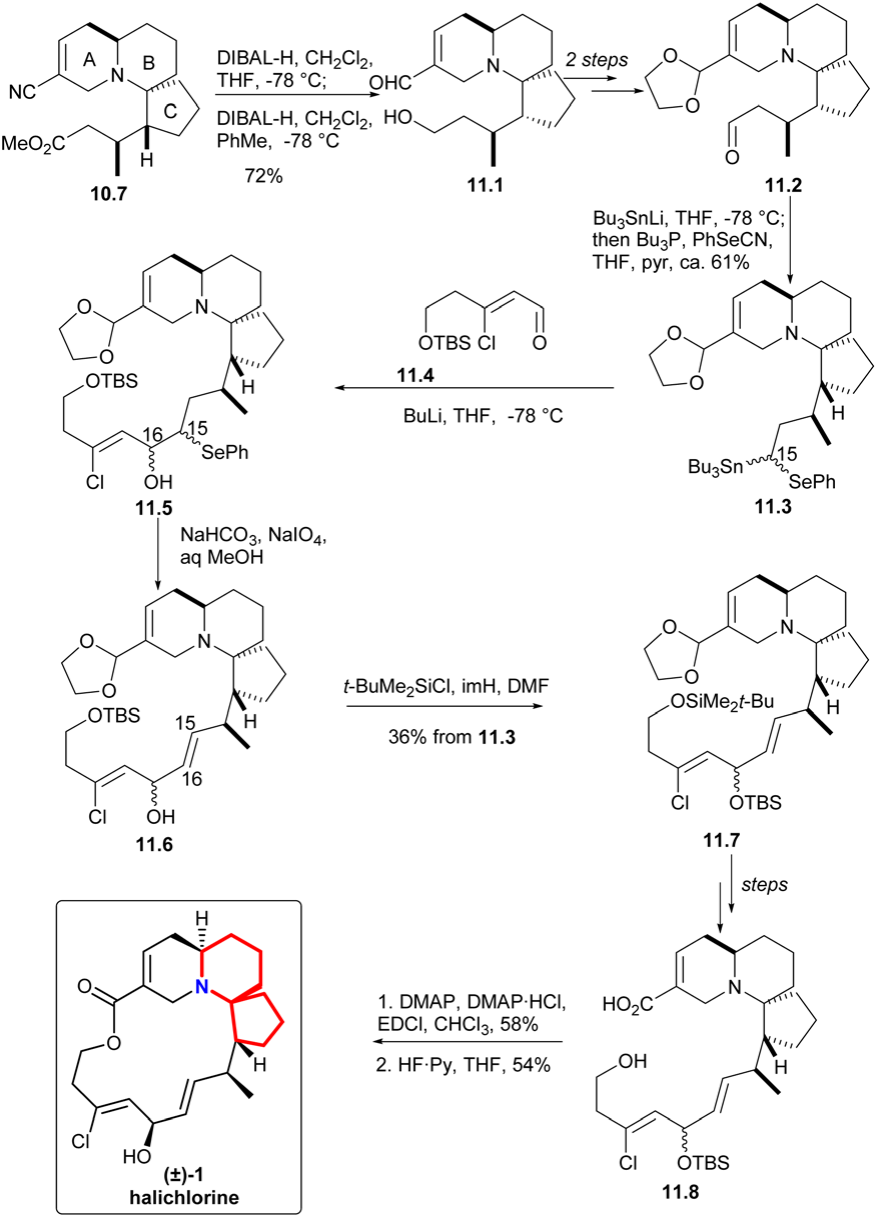
Clive's total synthesis of (±)-halichlorine.

In the presence of DIBAL-H, nitrile can be reduced to imine, which was then hydrolyzed and reduced to eventually yield alcohol 11.1 (Scheme 11). The processes of multiple steps could afford the compound aldehydes 11.2. By now the research group hoped to form a carboanion on C15 of halichlorine, which needed to produce selenium-stabilized carbanion and use selenium unit to make C15–C16 double bond. So as to, the stannyl alcohols mixture obtained by treating aldehydes with Bu_3_SnLi was immediately converted to the corresponding selenide 11.3. When the compound was treated with BuLi (to produce the desired selenium-stabilized carbanion^33^ by preferential C–Sn heterolysis) and then treated with the known compound 11.4,^6,34^ a mixture of β-hydroxyselenides 11.5 was obtained. The selenium was removed to liberate the double bond to give 11.6 during oxidation. Finally, the hydroxyl group was protected by silylation to obtain 11.7. By multi-step simple reactions, the hydroxyl and carboxyl groups of the compound 11.8 was cyclized by Keck macrocyclization and deprotected to form (±) halichlorine.

**Scheme 12:**
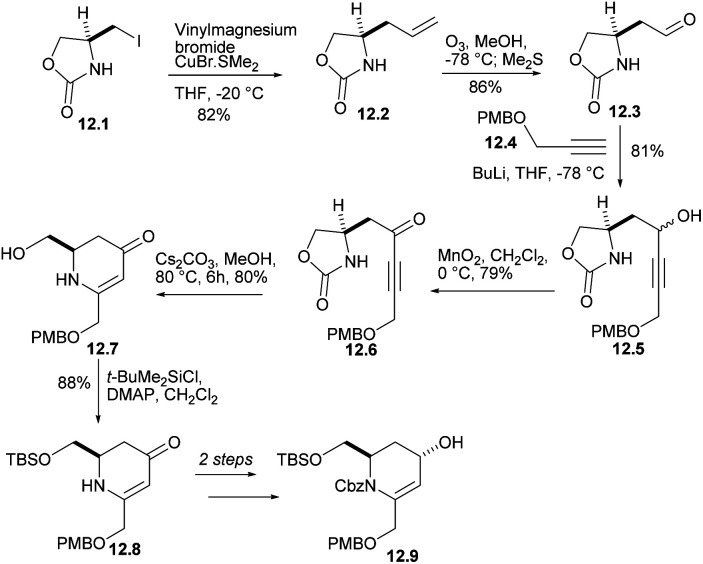
Synthesis of the precursor to the rearrangement substrate 12.9.

Iodide 12.1 was treated with lithium divinyl cuprate to produce 12.2 (Scheme 12). The terminal double bond of 12.2 performed ozone REDOX and reacted with compound 12.4 (ref. 35) under alkaline conditions to obtain the corresponding alcohols 12.5, which were oxidized to ketone 12.6. After treatment with Cs_2_CO_3_ in MeOH, ketone was converted into dihydropyridinone 12.7 at 80% yield. Each of the steps from l-serine to 12.7 occurred without loss of stereochemical integrity. After several reactions, alcohol 12.9 was obtained.

**Scheme 13:**
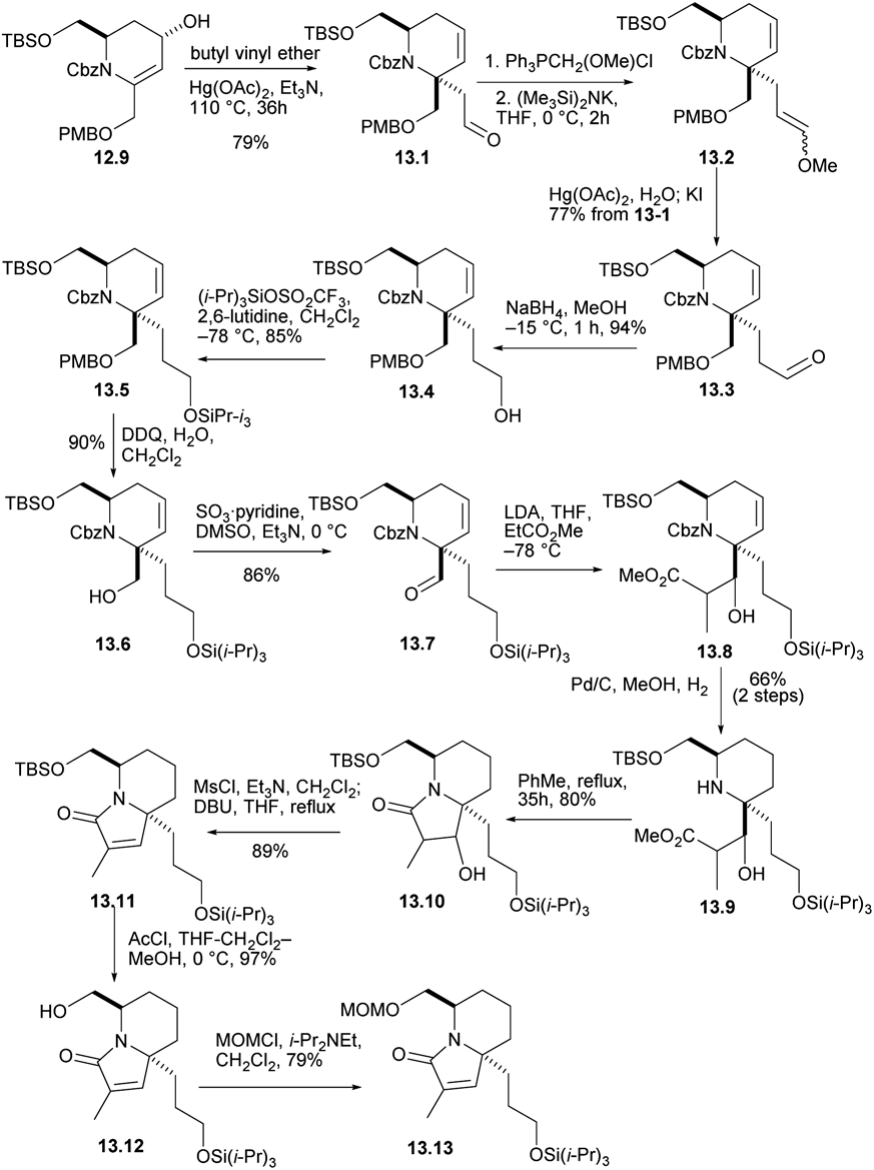
Preparation of optically pure intermediate.

The crucial claisen rearrangement reaction occurred when alcohol 12.9 was heated in butyl vinyl ether in the presence of Hg(OAc)_2_ and Et_3_N, which was finally converted to 13.1 with 79% yield (Scheme 13), and then Wittig enylation reaction occurred, and the enol ether 13.2 was converted into aldehyde 13.3. This was reduced and silylated (13.3 → 13.4 →13.5). The removal and oxidation of the PMB group gave aldehyde 13.6 and converted it to 13.7. Condensation of the enolate derived from EtCO_2_Me then gave conpound 13.8 and then reduced double bond. When these were heated for 35 h in boiling toluene, the lactam 13.10 were formed in good yield. Mesylation and heating with DBU in THF then converted compound 13.11. Next, the silicon masking the primary hydroxyl was removed with AcCl and the resulting alcohol 13.12 was protected as its MOM ether 13.13. The formation of optically pure 13.13 constitutes a formal synthesis, based on racemic route, of (+)-halichlorine.

#### 2.2.5 Uemura's enantioselective total synthesis of (+)-halichlorine

**Scheme 14:**
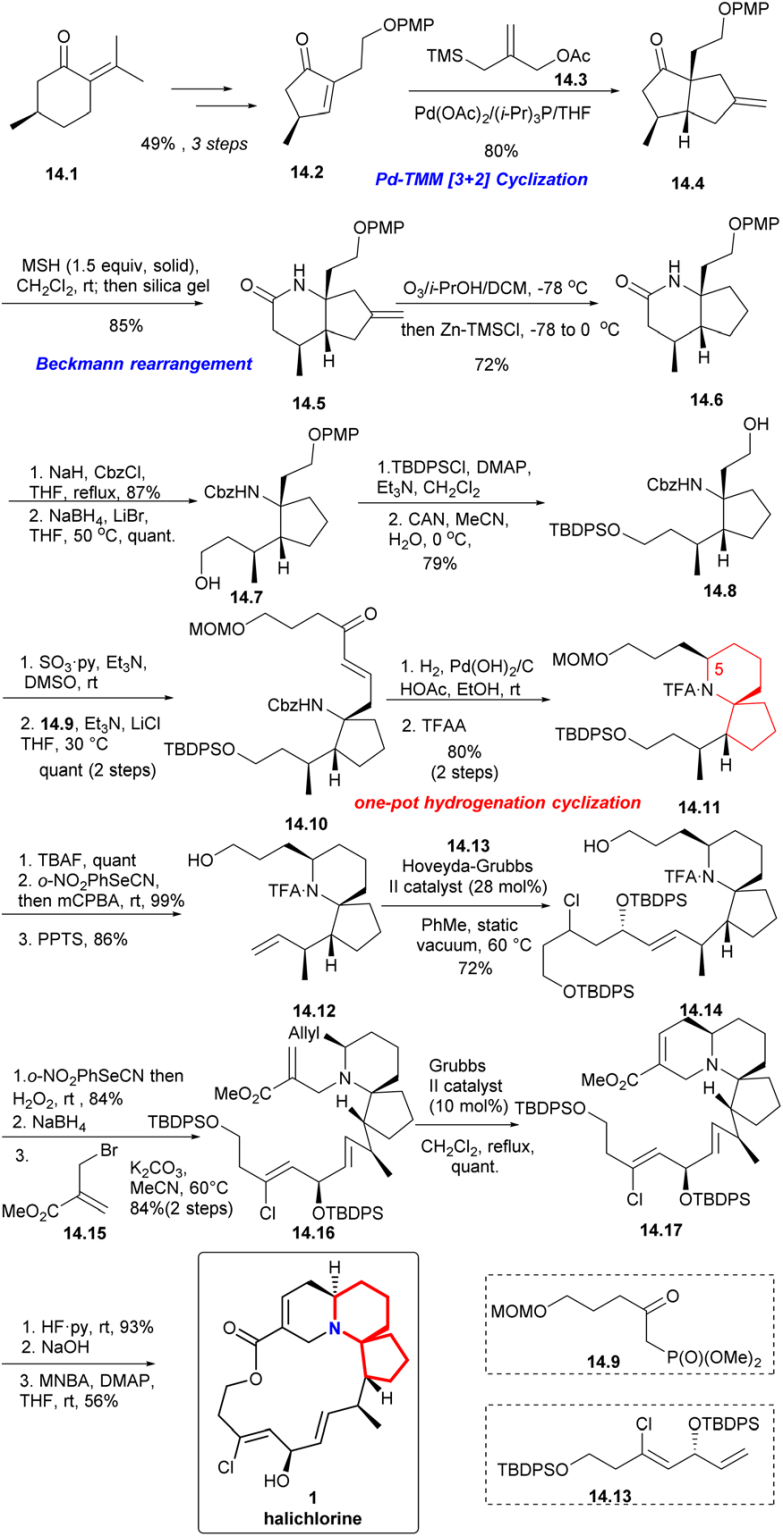
Uemura's enantioselective total synthesis of halichlorine.

In 2007, Uemura's group completed the asymmetric total synthesis of pinnaic acid.^36^ Then, they continued this route and completed the enantioselective total synthesis of halichlorine based on early result. In this section, we give a detailed report on this total synthesis route of (+)-halichlorine (Scheme 14).^37^

With (*R*)-pulegone 14.1 as the starting material, enone 14.2 obtained through 3 steps in 49% yield. Compound 14.2 reacted with the TMM^38^ precursor 14.3 highly selectively and only the diastereomer 14.4 was obtained in 80% yield. After Beckmann rearrangement^39^ and a one-pot ozonolysis-Clemmensen reduction process,^40^14.6 could be obtained in a high yield. Protection of 14.6 with Cbz and reduction with LiBH_4_ led to compound 14.7. Then, protection with TBDPS, removal of PMP *via* CAN, Parikh–Doering oxidation and the H–W–E reaction with 14.9 afforded spiro-cyclization precursor 14.10 as the single *trans* isomer (14.7 → 14.8 → 14.10). Next, using a one-pot hydrogenation-cyclization^41^ efficiently constructed the 6-azaspiro[4.5]decane skeleton with the desired C5 configuration and then TFA protected the amino group to obtained 14.11 through 2 steps in 80% yield. Selective removal of TBS, Grieco elimination^30^ and removal of MOM led to terminal olefin (14.11 → 14.12). The cross metathesis of 14.12 then proceeded smoothly with the lower-chain unit 14.13 under static vacuum conditions to afford compound 14.14 in 72% yield. Then, 14.14*via* 3 steps including Grieco elimination,^30^ deprotection of TFA and alkylation with bromo-substituted upper-chain unit 14.15 gave compound 14.16 in 84% yield.^42^ Six-membered RCM of 14.16 under the conditions reported by Kibayashi *et al.*^12*b*^ afforded the tricyclic compound 14.17 quantitative. Deprotection of the TBDPS groups, basic hydrolysis of the methyl ester and Shiina macrolactonization in overall 56% yield completed the total synthesis of (+)-halichlorine (14.17 → 1).

#### 2.2.6 Carreira's formal synthesis of (+)-halichlorine

In 2021, enantioselective and chemoselective iridium-catalyzed *N*-allylation of oximes^43^ were described for the first time by the Carreira's Laboratory. The realization of the *N*-allylation/1,3-dipolar cycloaddition reaction sequence provides the azaspirocyclic core in a highly enantioselective and diastereoselective manner. The efficient formal synthesis of Marine natural product (+)-halichlorine demonstrated the synthetic utility of this method.^44^

**Scheme 15:**
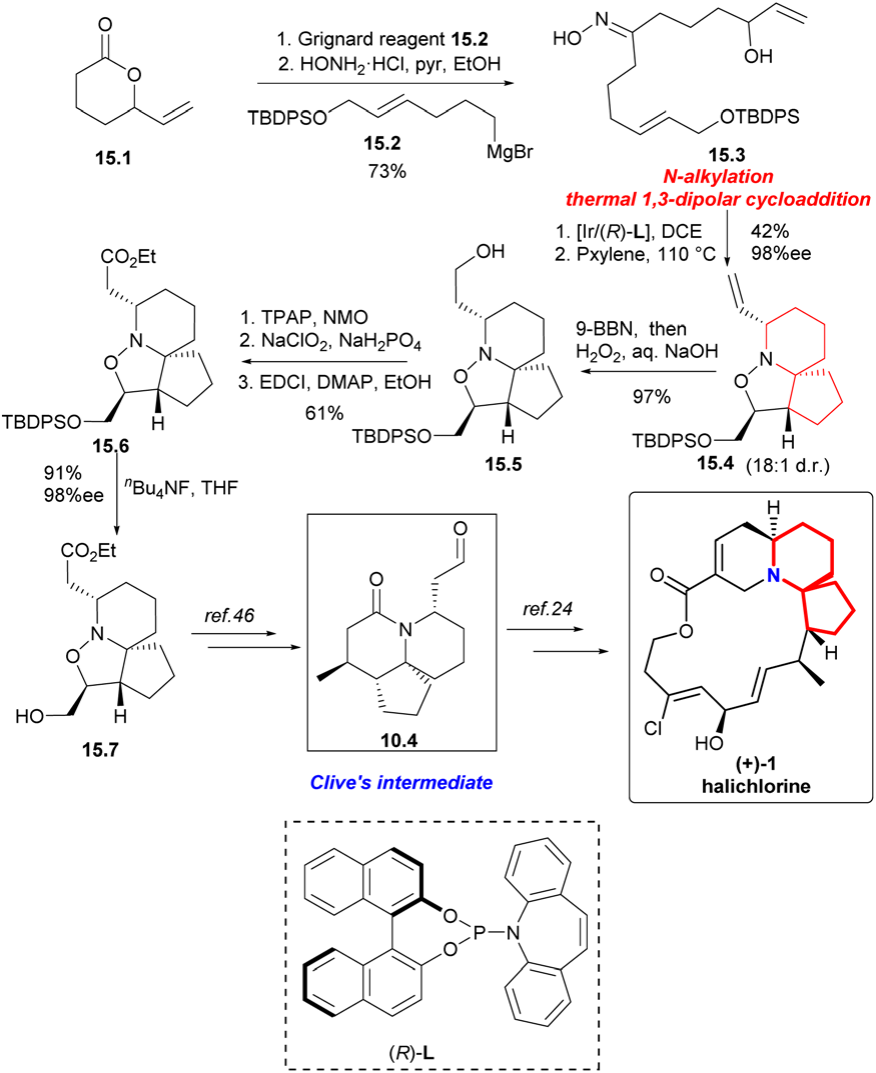
Carreira's formal synthesis of (+)-halichlorine.

Synthesis began with the Grignard addition reaction of 15.2 (Scheme 15) and racemic lactone 15.1.^45^ After treatment with hydroxylamine hydrochloride, oxime 15.3 was obtained by two-step separation. Iridium-catalyzed chemoselective *N*-alkylation and thermal 1,3-dipolar cycloaddition reactions provided compound 15.4 with four stereogenic centers with a yield of 42%, 98% ee and 18 : 1 d.r. on 2.5 mmol scale. The compound 15.4 was hydroborated and oxidized to the corresponding primary alcohol 15.5. The sequence of esterification by Ley oxidation, Pinnick oxidation, and Steglich provided a pathway for ester 15.6 to act as a single diastereomer. Finally, the silyl ether was cracked to obtain alcohol 15.7 with 91% yield,^46^ completing the synthesis of (+)-halichlorine.

## 3. Absolute configuration determination and asymmetric synthesis of pinnaic acid

### 3.1 Absolute configuration determination of pinnaic acid

**Scheme 16:**
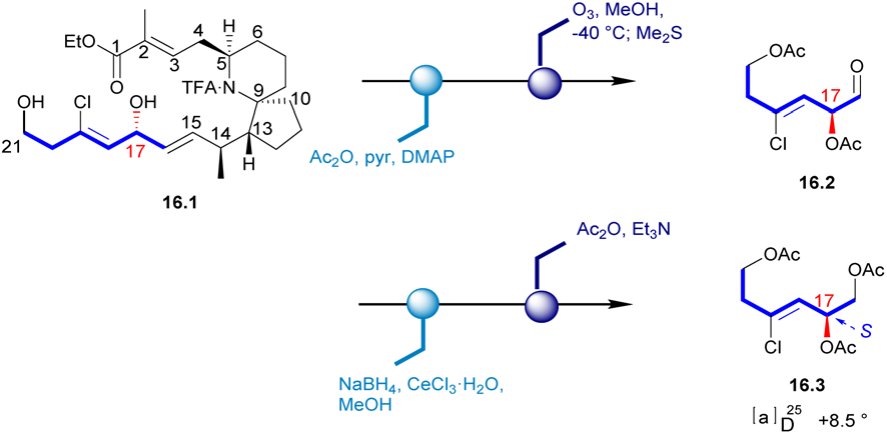
Confirmation of C17 stereochemistry of pinnaic acid.

In 1996, Uemura's Laboratory isolated pinnaic acid from the Okinawan bishell *Pinna muricata*, which was structurally closely related to halichlorine. Since only 1 mg of pinnaic acid was isolated from 3000 individual specimens of Okinawa bishell (*Pinna muricata*), it was not possible to correctly establish the stereochemistry of its structure in this case. Until 2001, Danishefsky's Laboratory determined the absolute configuration of pinnaic acid through synthetic method and clarified the stereochemistry of C14 and C17 chiral centers.^47,48^ The absolute configuration of the chiral center at C17 was established by degradation studies (Scheme 16). After acetylation of the key intermediate 16.1, the desired fragment was extracted through ozonolysis, resulting in aldehyde 16.2. Reduction and final acetylation of 16.2 gave the known compound 16.3 with the absolute configuration determined at C17. This correlation confirmed the absolute configuration of pinnaic acid and thus established the 17*R* configuration, as depicted in (and 2). The confirmation of the C14 configuration would be seen in subsequent Danishefsky's route.

### 3.2 Asymmetric synthesis of pinnaic acid

**Scheme 17:**
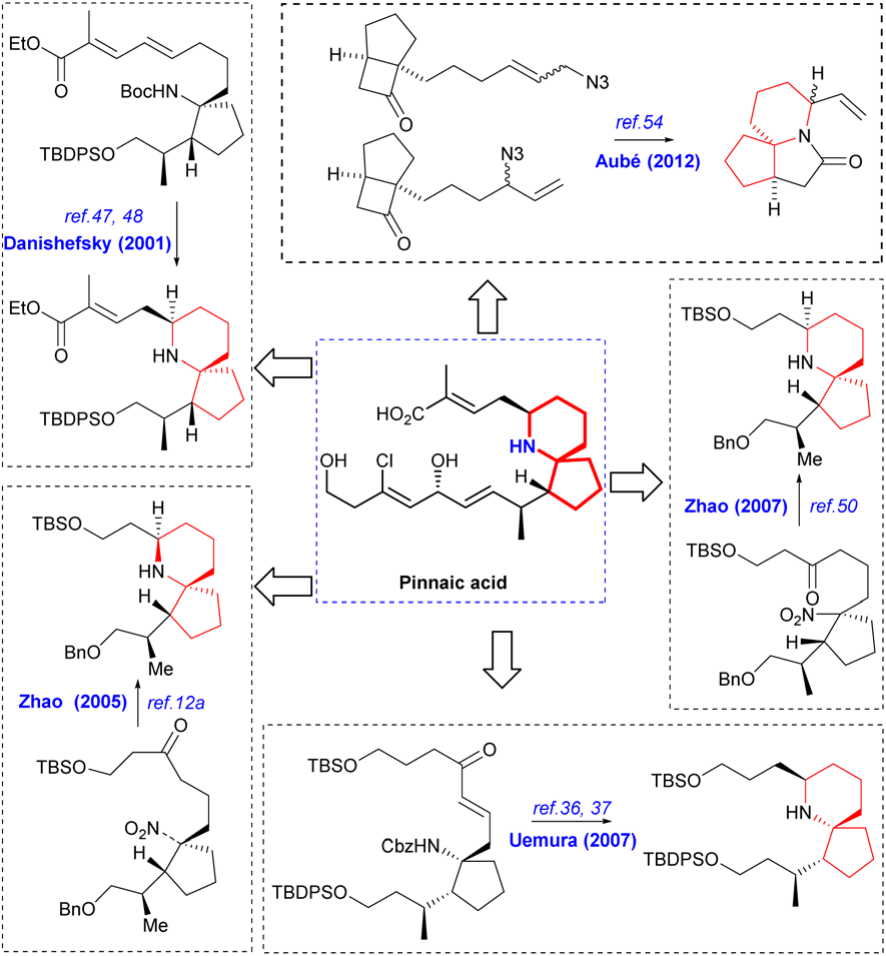
Asymmetric synthesis of pinnaic acid *via* the 6-azaspiro[4.5]decane skeleton.

The synthesis of pinnaic acid was initially accomplished by Danishefsky in 2001, and this achievement held reference significance for subsequent researchers working on synthesis. Other research groups have also successfully synthesized the pinnaic acid by constructing the 6-azaspiro[4.5]decane skeleton as the key steps through different strategies (Scheme 17).

#### 3.2.1 Danishefsky's first total synthesis of pinnaic acid

Due to the small amount of pinnaic acid obtained by isolation, the absolute configuration of the compound at C14 and C17 cannot be completely determined. Therefore, Danishefsky conducted a study on the synthesis of all four diastereoisomer derivatives.^47,48^

**Scheme 18:**
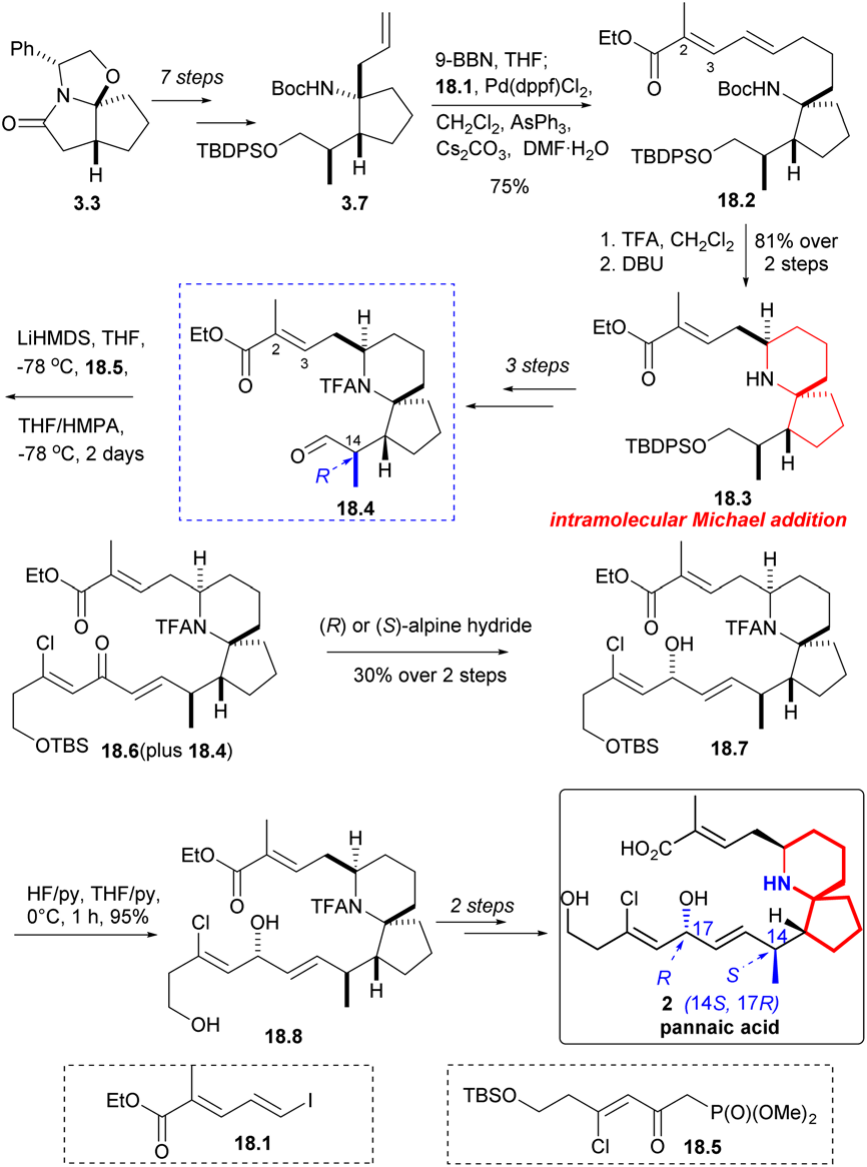
Danishefsky's synthesis of pinnaic acid and confirmation of C14 stereochemistry.

Compound 3.7 was obtained by a multi-step reaction starting with ‘Meyers lactam’ 3.3, which was then borohydrided again, followed by coupling with iodide 18.1 in the presence of palladium(ii) catalyst to obtain 18.2 (Scheme 18). Removal of the Boc group and treatment with DBU led to clean and stereoselective cyclization while retaining the *E* geometry of the resulting olefin 18.3. After three steps, the compound 18.4 C(14) isomer was synthesized. The aldehyde 18.4 underwent a H–W–E reaction using known phosphonate 18.5 (ref. 34) to generate α, β -unsaturated ketone 18.6. The reaction was incomplete and it was challenging to separate the desired product from the initial aldehyde 18.4. When the mixture of 18.4 and 18.6 was reduced by Alpine hydride,^49^ the resulting alcohol 18.7 exhibited the desired C17 configuration, irrespective of whether chiral *R* or *S*-hydride was used. Desilylation (18.7 → 18.8) and few steps reaction led to natural pinnaic acid.

**Scheme 19:**
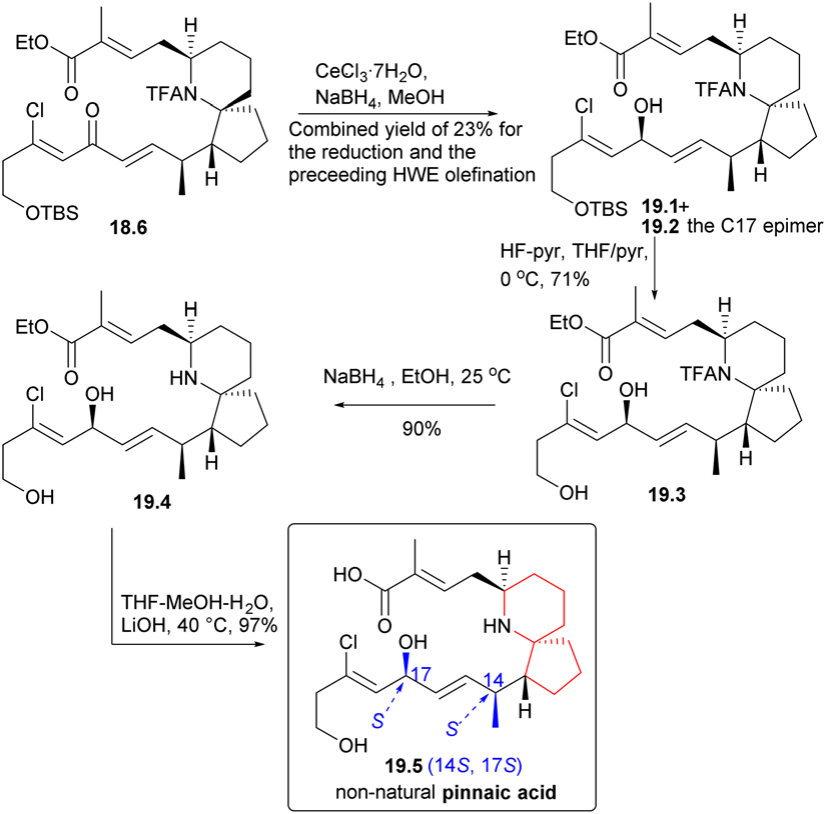
Danishefsky's synthesis of non-natural pinnaic acid.

During the course of the study, it was discovered that ketone 18.6 predominantly yielded alcohol 19.1 (Scheme 19) and its C17 epimers, alcohol 19.2, when subjected to Luche reduction conditions. Desilication, *N*-acetyl cleavage, and final ester hydrolysis yielded the non-natural pinnaic acid (19.2 → 19.3 → 19.4 → 19.5), whose 1HNMR spectrum was different from that of the natural compound.

**Scheme 20:**
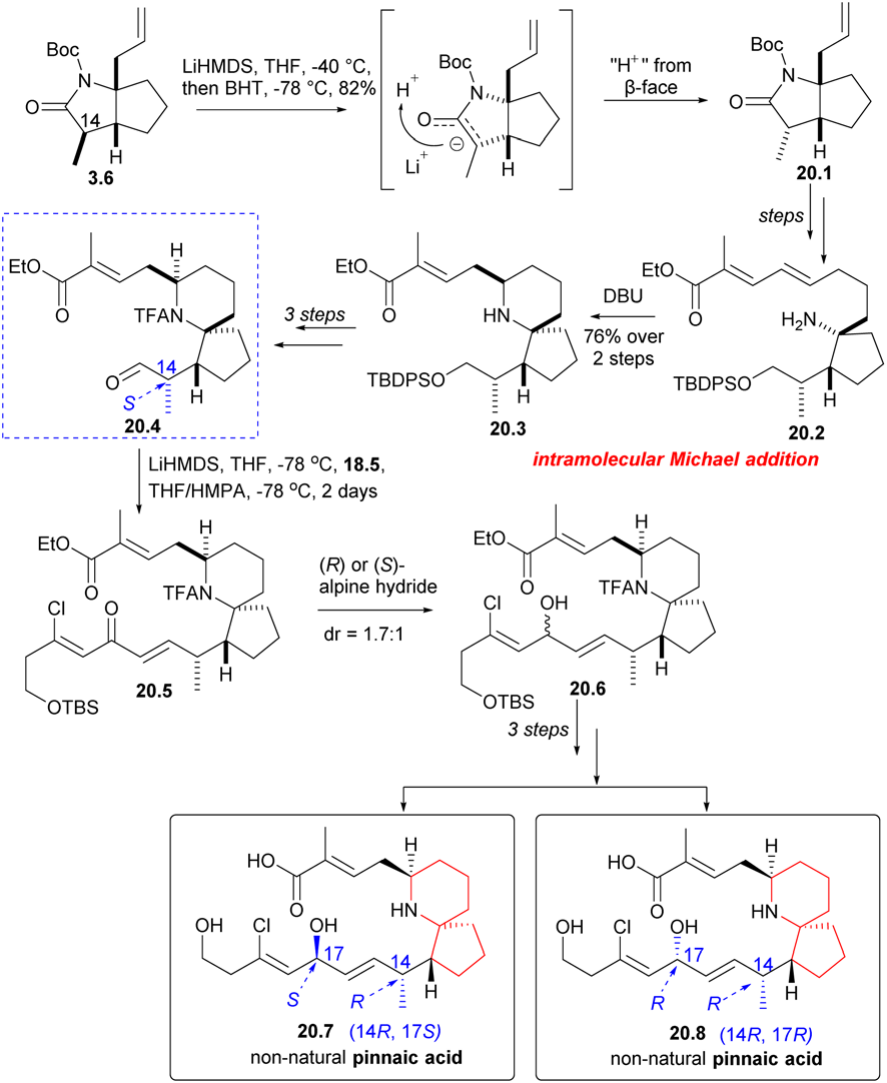
Danishefsky's synthesis of non-natural pinnaic acids.

Next, Danishefsky intended to prepare the C14 epimer of aldehyde 18.4. The route began with intermediate 3.6, which was deprotonated using LiHMDS and then re-deprotonated using BHT. This process led to a significant inversion of stereochemistry at the final C(14) position (3.6 → 20.1). The subsequent reactions followed the same procedure as described earlier, and the precursor compound 20.2 of the azabicyclic core was synthesized through a series of straightforward reactions. When treated with DBU, Micheal addition occurred to obtain the 6-azaspiro[4.5]decane skeleton 20.3, and aldehyde 20.4 was obtained after several steps reactions. The aldehyde 20.4 and phosphonate 18.5 underwent H–W–E reaction in the presence of lithium anion (20.4 → 20.5). Hydride reduction was employed once more, but this time without selectivity, resulting in a mixture of 20.6 and exomer alcohol in a ratio of 1.7 : 1. The diastereomers were subsequently isolated and transformed into non-native pinnaic acids 20.7 and 20.8 (Scheme 20).

After obtaining four diastereomers (2, 19.5, 20.7, 20.8), only 2 exhibited spectra that matched the spectral data of native pinnaic acid. The final task was to confirm the absolute configuration of C17, which was accomplished through degradation studies (see: Scheme 16).

#### 3.2.2 Zhao and Ding's formal synthesis of pinnaic acid

In 2005, Zhao and Ding successfully synthesized the key intermediate 5.16,^12*a*^ thereby completing the formal synthesis of halichlorine (as described in our previous summary of asymmetric halichlorine synthesis, see: Scheme 5). Following this path, they further synthesized another intermediate 21.3, which possessed an upper side chain, to achieve the formal synthesis of pinnaic acid.

**Scheme 21:**
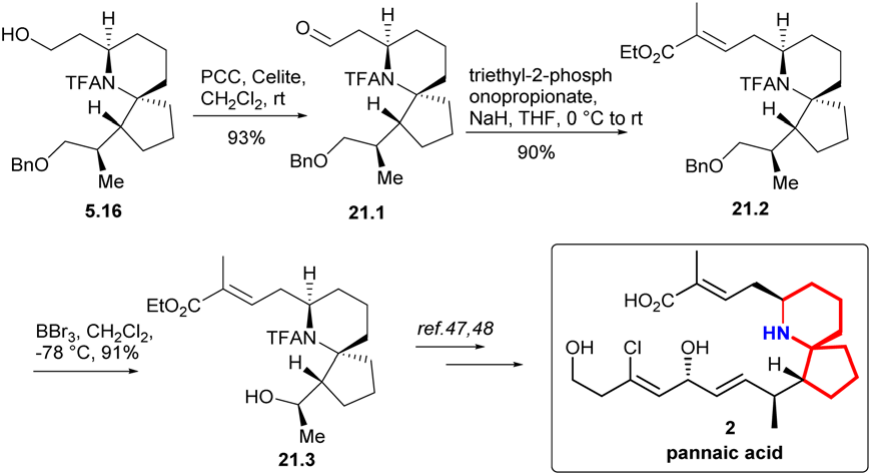
Zhao and Ding's formal synthesis of pinnaic acid.

Compound 21.1 was obtained oxidation of PCC (Scheme 21). Based on 21.1, they extended its side chain by alkenization. After H–W–E enylation, they constructed (*E*)-C2=C3, which was labelled as 21.2. and then alcohols were obtained by debenzoylation. The key intermediate 21.3 in Danishefsky route was obtained successfully.

The work of Zhao’ s group further enriches the research on the synthesis of halichlorine and pinnaic acid. They presented a novel and efficient method for the highly stereoselective synthesis of azospira rings using mild conditions.

#### 3.2.3 Zhao's enantioselective total synthesis of (−)-pinnaic acid

In 2005, Zhao's group completed the enantioselective synthesis of the 6-azaspiro[4.5]decane,^12*a*^ and one year later, they performed the enantioselective total synthesis of pinnaic acid.^50^ In this route, they improved the construction of azaspirocyclic core.

**Scheme 22:**
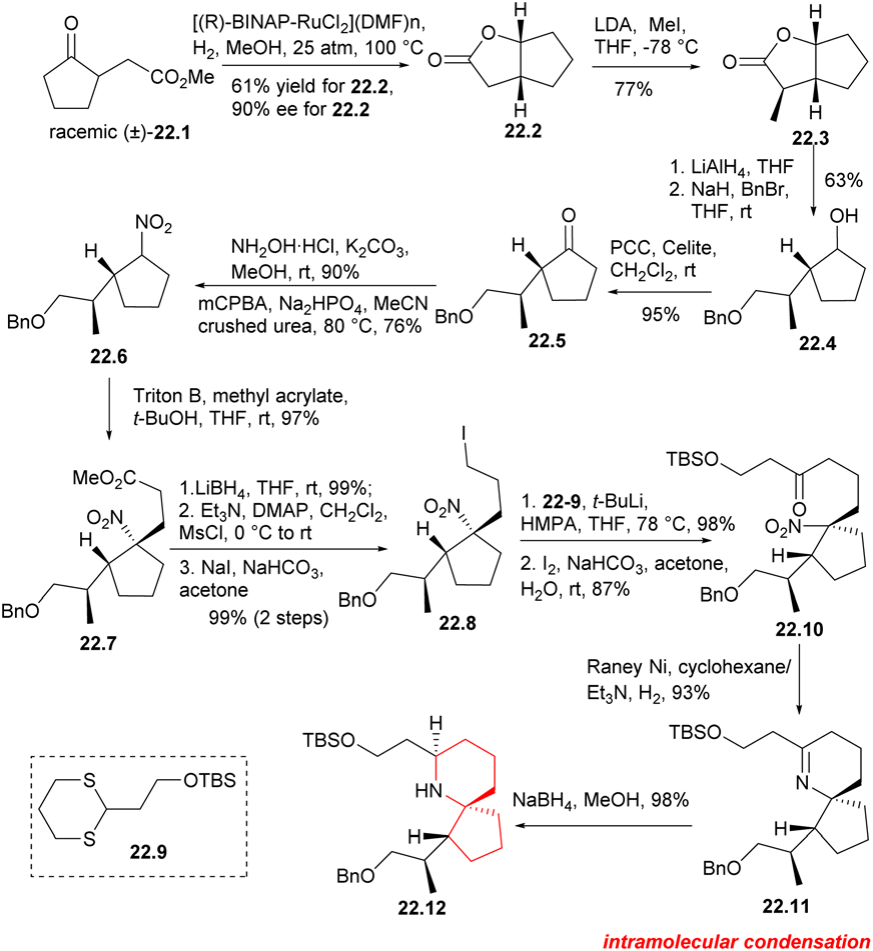
Construction of the azaspirocyclic core.

The BINAP–Ru complex has been found to be an efficient catalyst for the asymmetric hydrogenation of β-keto ester.^51^ In this synthesis, the catalyst was utilized to hydrogenate γ-ketone ester 22.1 (Scheme 22), resulting in the successful production of bicyclolactone 22.2 with satisfactory results. The asymmetric hydrogenation of racemic γ-keto ester 22.1 was conducted using [(*R*)-BINAP-RuCl_2_](DMF)n] as a catalyst. The desired (1*R*,5*R*)-lactone 22.2 was obtained in gram scale with a yield of 61% and an enantiomeric excess (ee) of 90%. The yield of the required lactone 22.2 was found to be higher than 50%, suggesting some deracemization of the α-position of the carbonyl group occurred under the reaction conditions. Asymmetric methylation of dicyclolactone 22.2 was carried out at −78 °C using LDA as a non-nucleophilic base, resulting in the formation of 22.3. LAH was used for reduction, followed by position-selective protection of primary alcohols with benzyl bromide. The secondary alcohols were then oxidized in CH_2_Cl_2_ using PCC to obtain the desired cyclopentanone 22.5. Hydroxylamine was condensed with cyclopentanone and then oxidized using *m*-CPBA to give nitrocyclopentane 22.6 with a yield of 64% in two steps.^52^ Compound 22.6 was a pair of diastereoisomers without further separation. The Michael addition reaction of nitrocyclopentane with methyl acrylate provided the foundation for the construction of the spirocenter. The desired nitroester 22.7 was obtained with 97% yield as a single diastereoisomer,^12*a*^ which can be explained by assuming that the acrylate approximated the less hindered face of the nitrocyclopentane. The nitroester was reduced with LiBH_4_, followed by mesylation and iodination to obtaine iodide 22.8. Subsequently, dithiane 22.9 was subjected to metallization using *t*-BuLi,^14^ and then alkylated with iodide 22.8.^12*a*,53^ Dithiane was then deprotected in the presence of I_2_ and NaHCO_3_ in acetone to obtaine ketone 22.10, which served as a crucial precursor for the cyclization of the piperidine ring. The nitro group in 22.10 was reduced to an amino group using RANEY^®^ Ni and H_2_ at 1 atmosphere pressure, and then the cycloimine compound 22.11 was formed through a simple nucleophilic reaction involving its carbonyl group. Nitrogen spiral ring 22.12 was successfully constructed by reducing cycloimine compound with NaBH_4_ in mixed solvent.

**Scheme 23:**
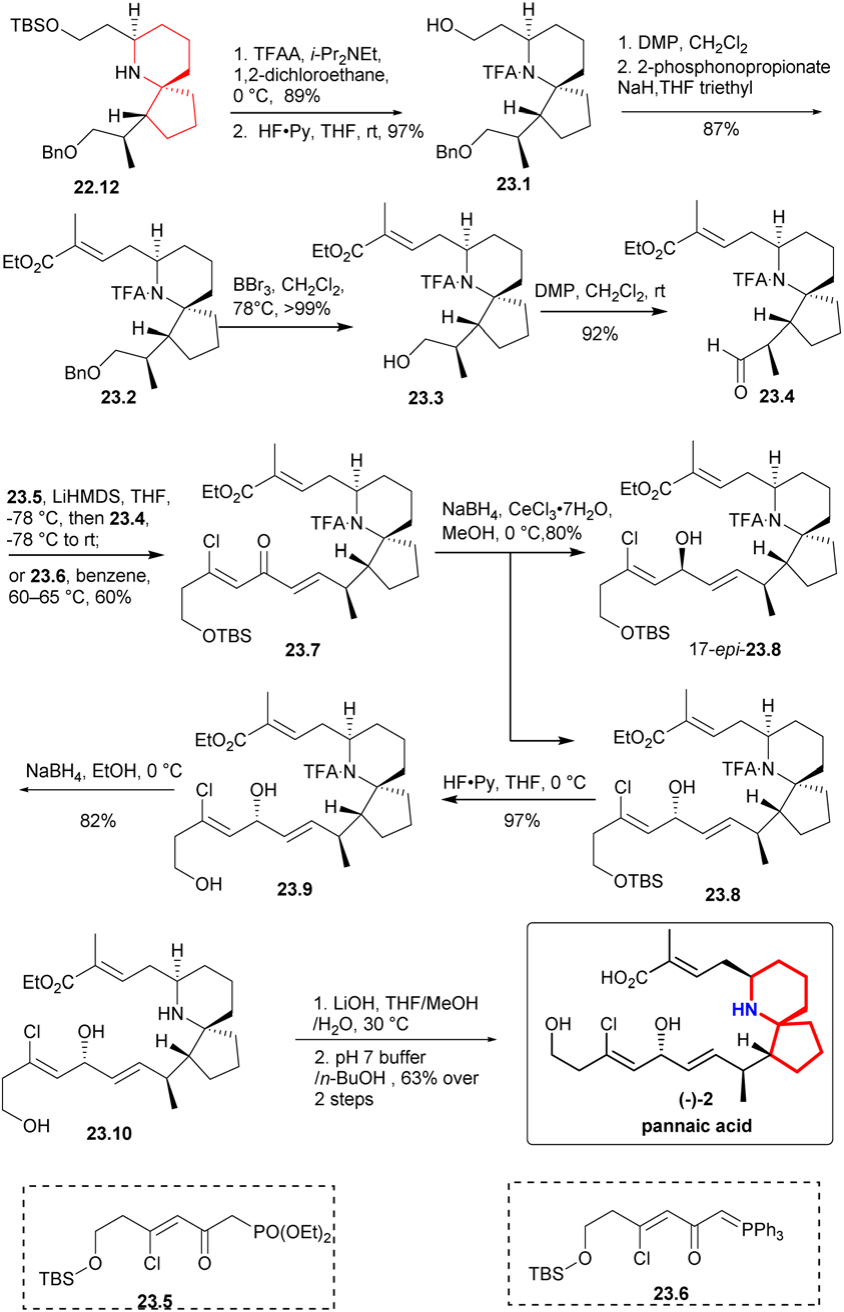
Zhao's total synthesis of pinnaic acid.

The secondary amino group was protected by TFAA,^47,48^ the TBS group was deprotected to obtain 23.1 (Scheme 23), and the aldehyde was oxidized with DMP and converted into compound 23.2 through H–W–E reaction. Subsequently, benzyl was removed using BBr_3_ at −78 °C, the resulting compound was then subjected to DMP oxidation, resulting in the desired aldehyde 23.4.and DMP oxidation yielded the desired aldehyde 23.4. A H–W–E reaction was performed between aldehyde 23.4 and Weinreb's phosphonate^34^23.5 to produce trace products. The desired dienone 23.7 was obtained at a moderate yield (60%) by heating aldehyde 23.4 and phosphorane 23.6 in benzene. Under the Luche reduction condition,^28^ the dienone was converted in a 3 : 1 mixture favoring the desired diastereomer 23.8. The TBS group was deprotected by HF-Py complex to produce the corresponding alcohol. To obtain lithium carboxylate salt of pinnaic acid, the trifluoroacetamide underwent reductive cleavage, followed by hydrolysis of the ethyl ester in the presence of LiOH. Pinnaic acid was synthesized enantioselectively by dissolving salt in an aqueous buffer solution of pH = 7, extracting it with 1-butanol and purifying it by liquid chromatography.

The highlight of this study was the use of a Ru complex as a catalyst for the asymmetric hydrogenation reaction, and facilitated the formation of the nitrogen azaspirocyclic core through multiple key reactions, leading to a shorter and more efficient synthesis route for pinnaic acid.

#### 3.2.4 Aubé’s asymmetric total synthesis of pinnaic acid

Aubé group combined the allyl azide rearrangement with the intramolecular Schmidt reaction to enantioselectively synthesize lactam 24.7,^54^ which was a key intermediate in the synthesis of (±)-pinnaic acid reported by Kibayashi *et al.*^12*b*^

**Scheme 24:**
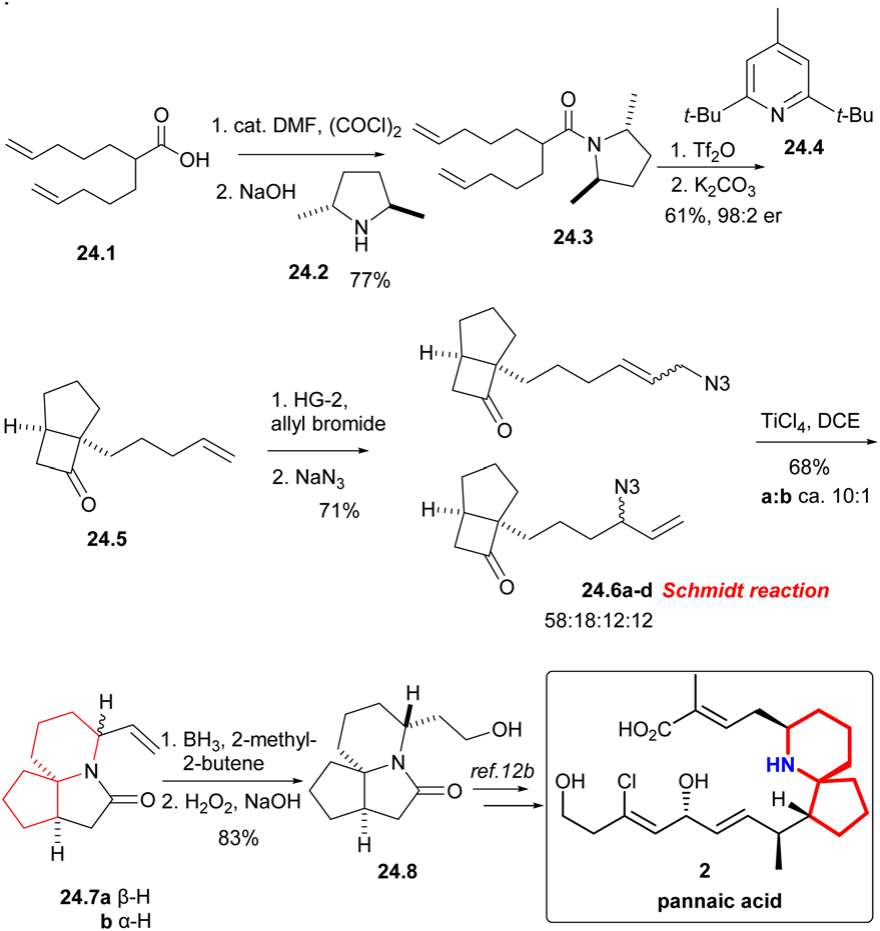
Aubé’s formal synthesis of pinnaic acid.

Under the condition of (COCl)_2_, the known acid 24.1 was converted into the corresponding acyl halide, and then ammonolysis occurred under basic conditions to obtain chiral amides 24.3 (Scheme 24). The asymmetric [2 + 2] cycloaddition of compound 24.3 using Ghosez's protocol,^55^ and the obtained intermediate iminium ions were alkaline hydrolyzed to cyclobutanone 24.5, which continued to undergo cross-metathesis,^56^ NaN_3_ displacement gave an interconverting mixture of allylic azides 24.6a–d. The Schmidt reaction of isomeric allyl azides, which was treated with TiCl_4_, was the key reaction, and the mixture of lactam 24.7a and 24.7b can be separated in a *ca.* 10 : 1 ratio. The stereochemistry of the reaction was controlled only by the position of the vinyl in the product. Finally, hydroboration/oxidation gave Kibayashi's pinnaic acid intermediate 24.8 in 83% yield.

#### 3.2.5 Uemura's asymmetric total synthesis of (−)-pinnaic acid

In 2007, the Uemura group described a novel strategy for asymmetric total synthesis using Pd-catalyzed trimethylenemethane [3 + 2] cyclization (Pd-TMM cyclization).^36^ In 2014, they further optimized this route, which achieved total synthesis of pinnaic acid.^37^

**Scheme 25:**
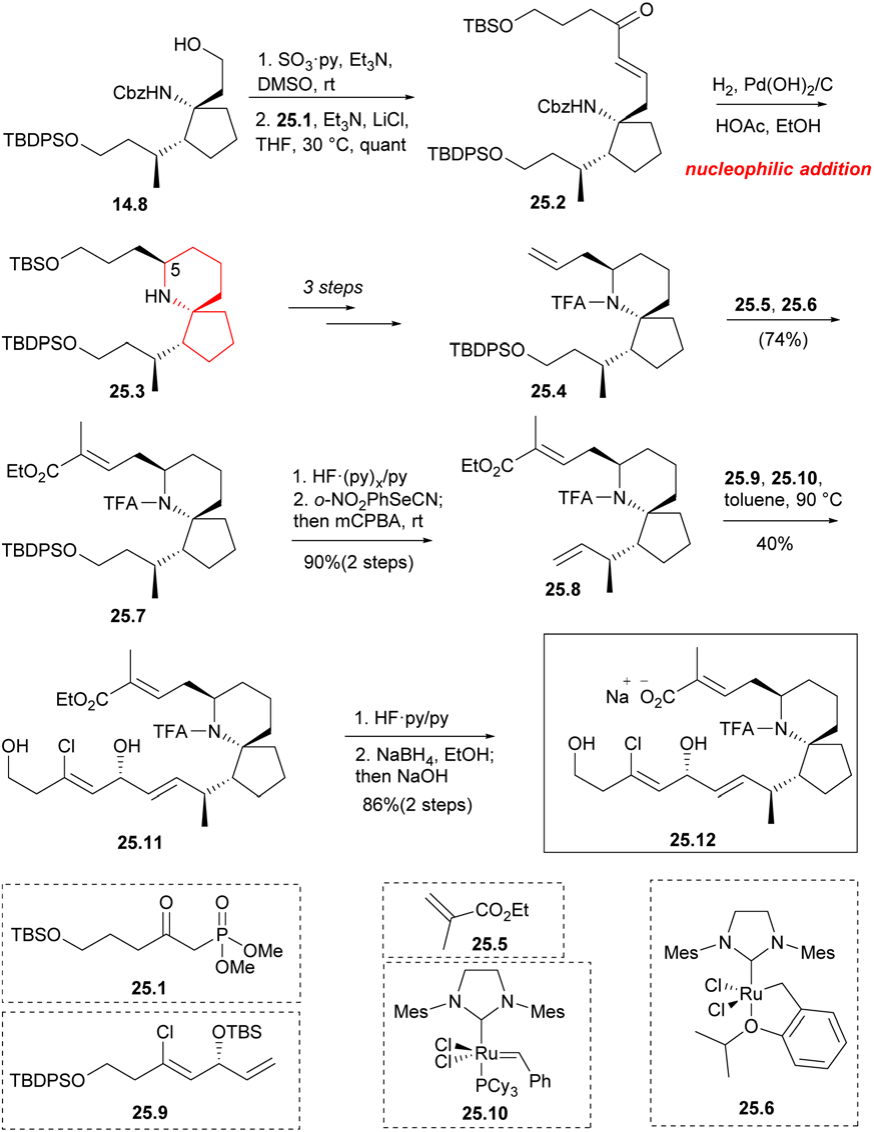
Uemura's total synthesis of (−)-pinnaic acid.

Aldehydes were formed by Parkin–Doering oxidation (SO_3_·Py, DMSO) from the key intermediate 14.8 and reacted with phosphonate 25.1 (Horner–Wadsworth–Emmons reaction). The product 25.2 was dominated by *E* isomer. This was followed by a series of hydrogenation–cyclization to construct a piperidine ring 25.3 with the desired C5 configuration.^41^ It consisted of four successive one-pot transformations: reduction of alkene double bonds; Cbz protective group was removed; formation of intramolecular cyclic imine; stereoselective reduction of imine/enamine intermediates. After multiple steps of reaction, 25.4 was obtained. By combining compound 25.5 (Scheme 25) with 10 mol% Hoveyda–Grubbs second-generation catalyst 25.6 (ref. 57) under reflux conditions, they obtained compound 25.7 with a yield of 74% during the above metathesis. TBDPS deprotection and Grieco elimination^30^ were employed to produce terminal olefin 25.8. This strategy utilized olefin cross-metathesis to establish the C17 center (90% ee) of segment 25.9 before it was incorporated into the spirocyclic core 25.8. Compound 25.9, which underwent a six-step transformation, was synthesized from but-3-yn-1-ol. The next step involved repeating the olefin cross-metathesis process. However, in the two precursors 25.8 and 25.9, there were four different types of carbon–carbon double bonds. It was observed that the two terminal double bonds were more reactive, possibly due to spatial factors. This reactivity led to the formation of a single trans isomer 25.11. The next step performed the olefin cross-metathesis again.^58^ However, in the two precursors 25.8 and 25.9, there were four types of carbon–carbon double bonds. It was observed that the two terminal double bonds were more reactive, possibly due to spatial factors. This reactivity led to the formation of a single trans isomer 25.11. Compound 25.11 was successively deprotected by the two silyl protecting groups, the TFA amide, and the ethyl ester using common methods^48^ to obtain chiral pinnaic acid (25.12) in the form of sodium salt.

## 4. Racemic synthesis of halichlorine and pinnaic acid

In addition to Clive group's review,^3^ we also summarized the racemization work of halichlorine and pinnaic acid that was not included.

### 4.1 Martin's formal synthesis of pinnaic acid and halichlorine

Martin's group synthesized the known intermediates 26.9 and 27.5 to achieve the formal synthesis of halichlorine and pinnaic acid.^59^ This route effectively installed the upper chain through the key reaction: cross-olefin-metathesis,^60^ which illustrated the utility of selective olefin cross metathesis methodologies for the elaboration of advanced synthetic intermediates in complex molecule synthesis.

**Scheme 26:**
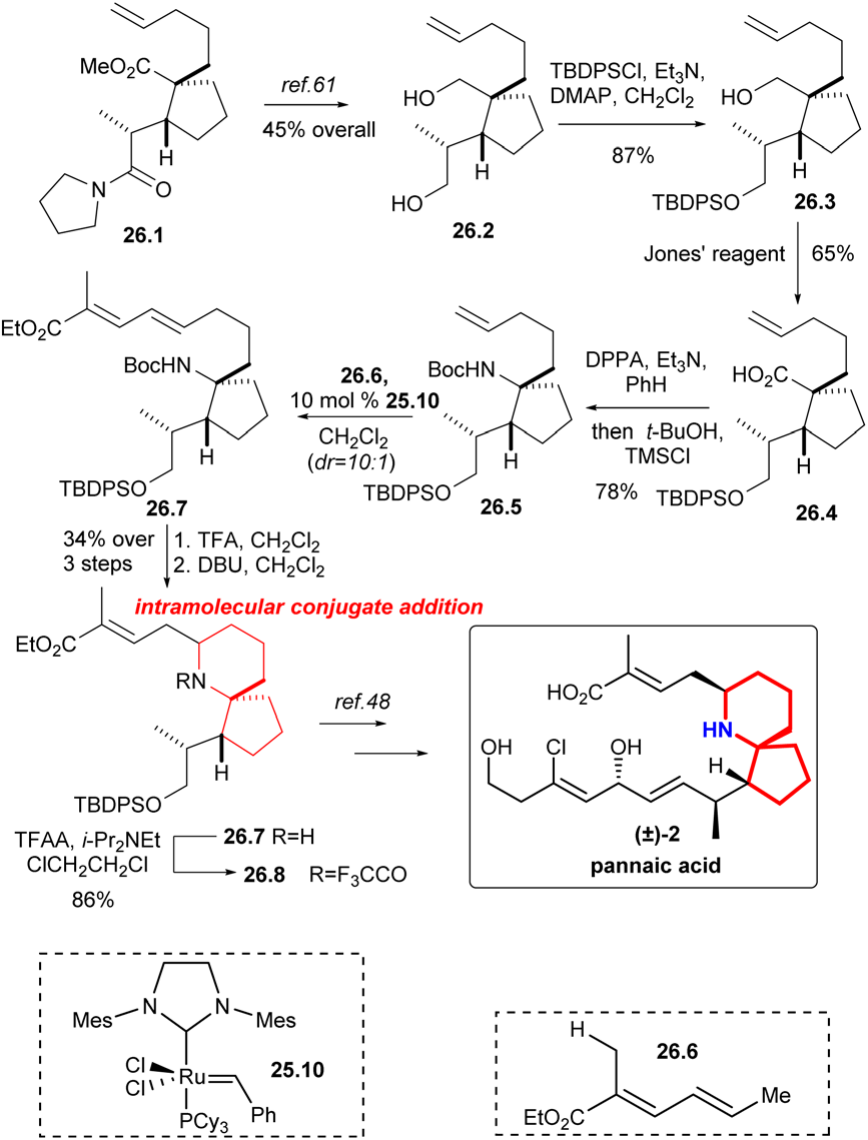
Martin's formal synthesis of (±)-pinnaic acid.

The synthesis commenced with the compound 26.1 (Scheme 26), which was converted to alcohol 26.2 by literature.^61^ The resulting primary alcohol 26.2 was protected as its *tert*-butyl-diphenylsilyl derivative 26.3. Oxidation with Jones' reagent then generated carboxylic acid 26.4 as expected, which was subjected to a Curtius rearrangement with DPPA^22^ and *t*-BuOH to afford 26.5. At this point cross metathesis of 26.5 with the ester 26.6, using the Grubbs II catalyst 25.10,^58^ produced the *E*-olefin 26.7, which upon deprotection of the amino function with TFA and cyclization of the intermediate amino dienoate *via* intramolecular 1,6-conjugate addition to afford piperidine 26.8 in 34% yield for the three steps. The nitrogen was protected as its trifluoroacetate 26.9, an intermediate in the Danishefsky's synthesis of pinnaic acid.^48^

**Scheme 27:**
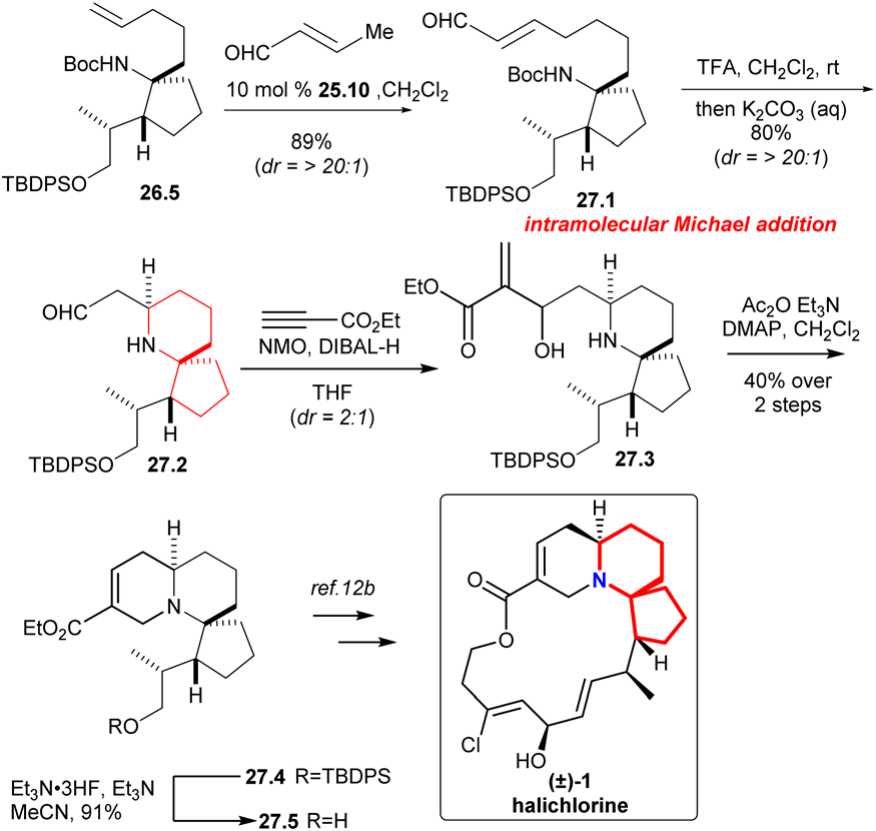
Martin's formal synthesis of (±)-halichlorine.

Compound 27.1 (Scheme 27) was obtained in 89% yield with excellent diastereoselectivity by compound 26.5 and 10 mol% Grubbs II catalyst 25.10.^58^ Carbamate cleavage facilitated spontaneous Michael addition affording spirocycle 27.2. The reaction of ethyl propiolate in THF with DIBAL-H/NMO complex provided the vinylaluminum reagent.^62^ Aldehyde 27.2 was added to the vinylaluminum reagent to gain compound 27.3 as a mixture of diastereomers (dr = 2 : 1). Acetylation of this mixture under standard conditions led to a facile cyclization that provided the known tricycle 27.4. Final desilylation provided ester 27.5, a substance synthesized previously by Kibayashi.^12*b*^

### 4.2 Clive's total synthesis of (±) halichlorine

(See 2.2.4).

### 4.3 Stockman's formal synthesis of halichlorine

In 2004, Stockman's group reported a concise method for preparing the azaspirocyclic core structure of halichlorine and pinnaic acid with a tandem cascade strategy.^63^ In 2012, they improved this strategy and completed the formal synthesis of halichlorine.^46^ The resulting synthesis of azaspirocyclic aldehyde 10.4 was an intermediate for the total synthesis of halichlorine by Clive's laboratory.^24^

**Scheme 28:**
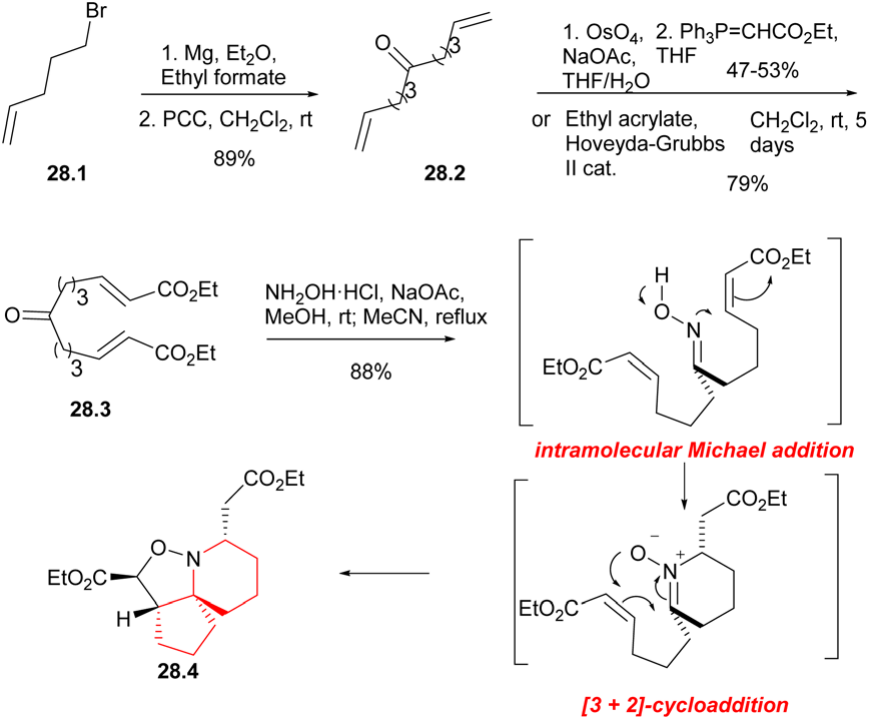
Synthesis of the 6-azaspiro[4.5]decane skeleton of halichlorine.

By utilizing an electrophilic centrepiece for the two-directional approach, symmetrical alcohol 28.1 (Scheme 28) was successfully synthesized through double addition of pent-5-enyl magnesium bromide on ethyl formate.^64^ The alcohol function of 28.1 was then oxidized, resulting in the formation of dialkene 28.2 with a high overall yield. Ketodiester 28.3 could through either the two-directional cross-metathesis method or a two-step approach involving oxidative addition of the alkenes of 28.2 followed by two-directional Wittig homologation. When performed on a 10 g scale, the two-step approach gave the ketodiester 28.3 in 47–53% yield after purification, and was found to be more cost-effective compared to the cross-metathesis route. Upon treatment with hydroxylamine hydrochloride in the presence of sodium acetate, symmetrical ketodiester 28.3 underwent a transformation the [6,5,5]tricycle 28.4 in 88% yield, this transformation was achieved through a tandem oxime formation/Michael addition/1,4-prototopic shift/[3 + 2]-cycloaddition.^65^

**Scheme 29:**
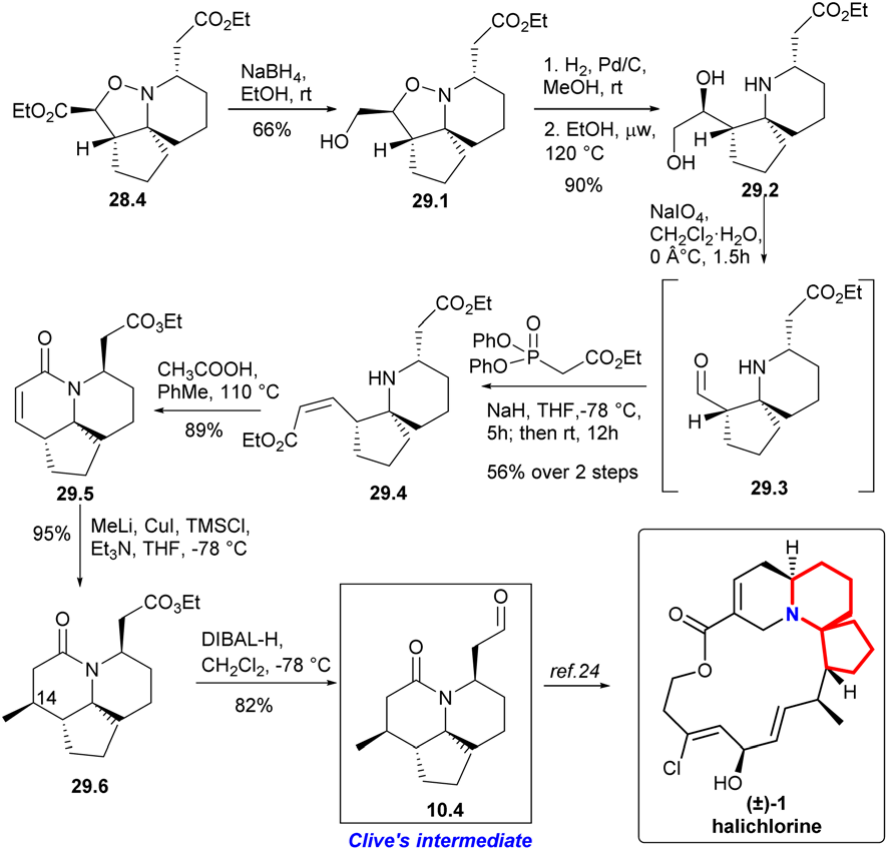
Stockman's formal synthesis of halichlorine.

The isoxazolidine 28.4 was selectively reduced to 29.1 in ethanol using sodium borohydride^64^ and subsequent hydrogenation cleaved the N–O bond, resulting in the formation of diol 29.2 with a quantitative yield (Scheme 29). Ester 29.4 with *Z*-conformation was produced by oxidative cleavage of diol followed immediately by an Ando homologation reaction.^66^ It was observed that the reaction yielded the best results when conducted in refluxing toluene with stoichiometric acetic acid, resulting in a high yield of lactam 29.5. Addition of the Gilman reagent^67^ to 29.5 afforded the lactam 29.6 in excellent yield. They found that this reaction required both TMSCl and triethylamine to proceed. With full stereoscopic control of C14 methyl, the ester functional group was converted to the target aldehyde 10.4 using DIBAL-H reduction at low temperature. So they had completed a short and efficient synthesis of Clive's aldehyde 10.4 in 12 steps and 13.2% overall yield from ethyl formate. This study represents the formal synthesis of halichlorine through the rapid and efficient synthesis of Clive aldehyde 10.4.^24^

### 4.4 Zhu's formal synthesis of halichlorine

In 2015, a Au^I^/Cu^II^-cocatalyzed tandem cyclization/semipinacol reaction^68^ had been developed by Zhu's group to provide an effective method for the construction of the 6-aza/oxa-spiro[4.5]decane skeletons. The synthetic utility of the approach was demonstrated by the efficient, formal synthesis of the marine natural product (±)-halichlorine.^69^

**Scheme 30:**
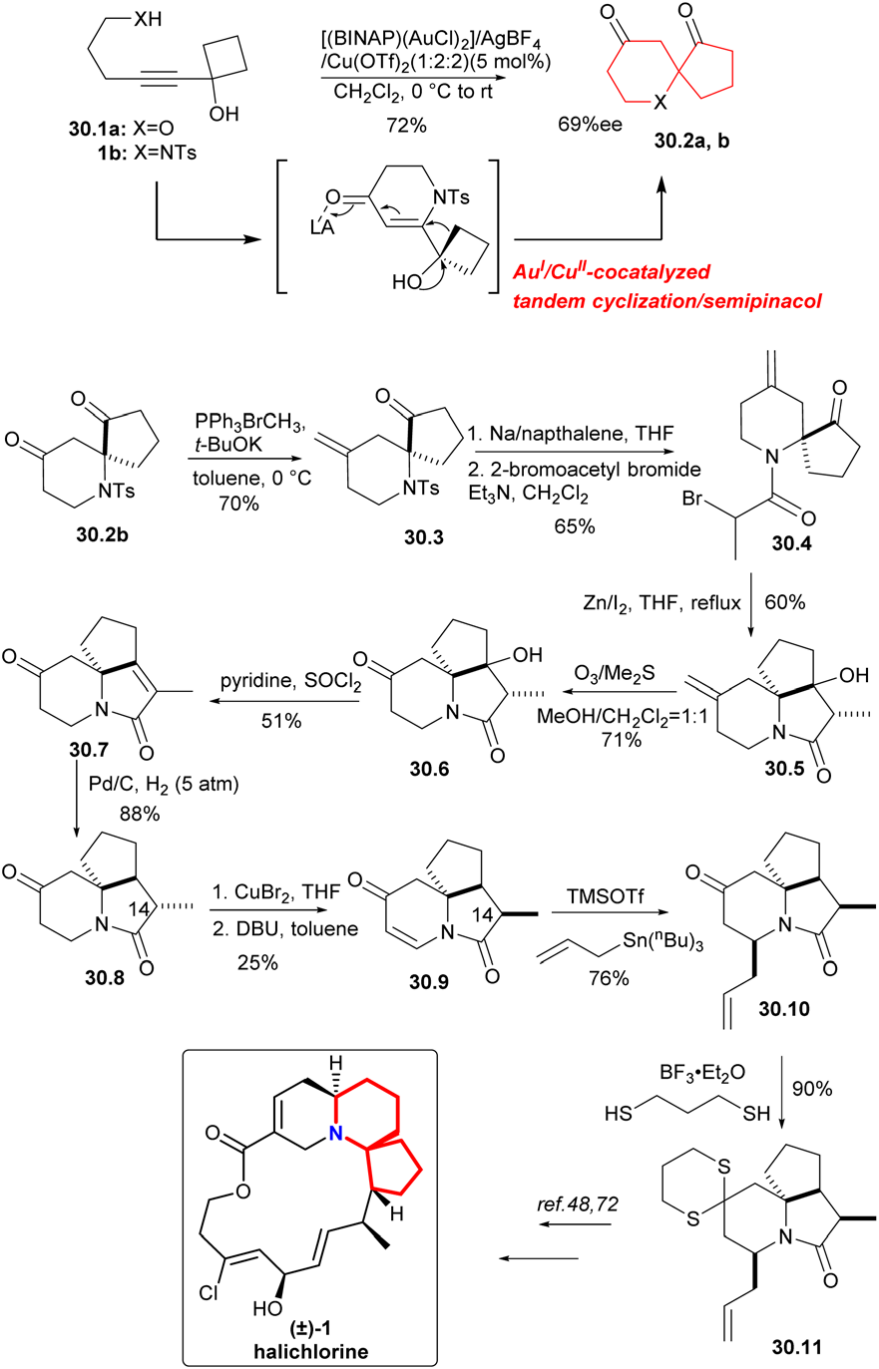
Zhu's formal synthesis of (±)-halichlorine.

The Au^I^/Cu^II^-cocatalyzed tandem cyclization/semipinacol reaction was conducted with substrate 30.1a as the model substrate. After trying many conditions, [(BINAP)(AuCl)_2_]/AgBF_4_/Cu(OTf)_2_(1 : 2 : 2) was selected as the catalyst to construct the 6-aza/oxa-spiro[4.5]decane skeletons 30.2a,b (Scheme 30).^70^ The aza-spiro-ketone 30.2b was subjected to Wittig olefination with Ph_3_PBrCH_3_ in order to protect the carbonyl group (because the carbonyl group on the piperidine ring would cause side reactions in the subsequent nucleophilic steps), followed by removing the Ts group and amidation with 2-bromoacetyl bromide (30.2b → 30.4). For the construction of tricyclic intermediate 30.5, the bromine substitute compound 30.4 was subjected to intramolecular Reformatsky reaction with iodine as the initiator in a 60% yield. Ozonolysis and reductive workup gave 30.6, and dehydration led to amide 30.7,^71^ which gave 30.8 by catalytic hydrogenation. Introduction of the desired carbon–carbon double bond into 30.9 was achieved using copper bromide and DBU, which can get α-bromination compound, and then followed by the elimination reaction. At the same time, the configuration reversion of the C-14 methyl, was also successfully achieved. Subsequently, intermediate 30.10 was obtained by a 1,4-addition of amide 30.9 with allylstannane. Reaction of 4-piperidonone 30.10 with 1,3-propane thiol gave in a 90% yield the expected dithiane 30.11, they had completed an advanced intermediate reported by Padwa's group,^72^ representing a formal synthesis of (±)-halichlorine.

## 5 Conclusions

In summary, we mainly focus on the construction of the 6-azaspiro[4.5]decane skeleton and provide many brief account of the asymmetric synthesis routes of two marine alkaloids: halichlorine and pinnaic acid. The unique structures and interesting bioactivity of pinnaic acid and halichlorine have motivated the exploration of a number of total syntheses. The synthesis of halichlorine and pinnaic acid was first accomplished by Danishefsky's research group in 1999 and 2001. This groundbreaking work holds great historic significance as it fills a gap in the total synthesis history of these compounds. In this review, we can see from the research works of various groups that the construction of C5, C9 and C13 three stereogenic carbons in the 6-azaspiro[4.5]decane skeleton is somewhat the challenging and key step. And different construction strategies of 6-azaspiro[4.5]decane frameworks of halichlorine and pinnaic acid have been also developed.^73^ In addition to the asymmetric synthesis methods summarized in this review, many research groups have also successfully completed the racemic synthesis of the halichlorine and pinnaic acid.^3,24,46,59,69^ These outstanding synthetic works make the two alkaloids more attractive. We hope that more relevant researchers will have the courage to challenge and synthesize halichlorine and pinnaic acid in a more concise and efficient way.

## Conflicts of interest

There are no conflicts to declare.

## Supplementary Material
